# Identification of TFPI as a receptor reveals recombination-driven receptor switching in *Clostridioides difficile* toxin B variants

**DOI:** 10.1038/s41467-022-33964-9

**Published:** 2022-11-09

**Authors:** Songhai Tian, Xiaozhe Xiong, Ji Zeng, Siyu Wang, Benjamin Jean-Marie Tremblay, Peng Chen, Baohua Chen, Min Liu, Pengsheng Chen, Kuanwei Sheng, Daniel Zeve, Wanshu Qi, David T. Breault, César Rodríguez, Ralf Gerhard, Rongsheng Jin, Andrew C. Doxey, Min Dong

**Affiliations:** 1 Department of Urology, Boston Children’s Hospital, Boston, MA 02115 USA; 2 Department of Microbiology and Department of Surgery, Harvard Medical School, Boston, MA 02115 USA; 3 School of Biomedical and Pharmaceutical Sciences, Guangdong University of Technology, Guangzhou, 510006 China; 4 Department of General Surgery, Sir Run Run Shaw Hospital, School of Medicine, Zhejiang University, Hangzhou, Zhejiang 310016 China; 5 Department of Biology, Cheriton School of Computer Science, and Waterloo Centre for Microbial Research, University of Waterloo, Waterloo, ON N2L 3G1 Canada; 6 Department of Physiology and Biophysics, School of Medicine, University of California Irvine, Irvine, CA 92697 USA; 7 Wyss Institute for Bioinspired Engineering, Harvard University, Boston, MA 02115 USA; 8 Division of Endocrinology, Boston Children’s Hospital, Boston, MA 02115 USA; 9 Department of Pediatrics, Harvard Medical School, Boston, MA 02115 USA; 10 Harvard Stem Cell Institute, 7 Divinity Avenue, Cambridge, MA 02138 USA; 11 Faculty of Microbiology & CIET, University of Costa Rica, San José, Costa Rica; 12 Institute of Toxicology, Hannover Medical School, 30625 Hannover, Germany

**Keywords:** Pathogens, Cellular microbiology, Bacterial pathogenesis, Bacterial toxins

## Abstract

Toxin B (TcdB) is a major exotoxin responsible for diseases associated with *Clostridioides difficile* infection. Its sequence variations among clinical isolates may contribute to the difficulty in developing effective therapeutics. Here, we investigate receptor-binding specificity of major TcdB subtypes (TcdB1 to TcdB12). We find that representative members of subtypes 2, 4, 7, 10, 11, and 12 do not recognize the established host receptor, frizzled proteins (FZDs). Using a genome-wide CRISPR-Cas9-mediated screen, we identify tissue factor pathway inhibitor (TFPI) as a host receptor for TcdB4. TFPI is recognized by a region in TcdB4 that is homologous to the FZD-binding site in TcdB1. Analysis of 206 TcdB variant sequences reveals a set of six residues within this receptor-binding site that defines a TFPI binding-associated haplotype (designated B4/B7) that is present in all TcdB4 members, a subset of TcdB7, and one member of TcdB2. Intragenic micro-recombination (IR) events have occurred around this receptor-binding region in TcdB7 and TcdB2 members, resulting in either TFPI- or FZD-binding capabilities. Introduction of B4/B7-haplotype residues into TcdB1 enables dual recognition of TFPI and FZDs. Finally, TcdB10 also recognizes TFPI, although it does not belong to the B4/B7 haplotype, and shows species selectivity: it recognizes TFPI of chicken and to a lesser degree mouse, but not human, dog, or cattle versions. These findings identify TFPI as a TcdB receptor and reveal IR-driven changes on receptor-specificity among TcdB variants.

## Abstract (web summary)

Toxin B (TcdB) is a major exotoxin responsible for diseases associated with *C. difficile* infection. Here, Tian et al. show that several TcdB subtypes do not recognize the established FZD receptors, and identify a different host protein (TFPI) as a receptor for subtypes TcdB4 and TcdB10.

## Introduction

*Clostridioides difficile* (formerly *Clostridium difficile*) is a Gram-positive bacterium with spores distributed widely in the natural environment^1,2^. Toxigenic strains of *C. difficile* have emerged as a major opportunistic pathogen to humans and animals since 1970s with the use of broad-spectrum antibiotics that disrupt gut microbiota. With over a quarter million cases in the United States annually, *C. difficile* infection (CDI) has become a leading cause of hospital-associated gastrointestinal infection^1–3^, and *C. difficile* is one of the most urgent antibiotic-resistant threats classified by the U.S. Center for Disease Control and Prevention^3,4^.

CDI can cause symptoms ranging from self-limiting diarrhea to life-threatening colitis and toxic megacolon, and 15–35% of patients may suffer from recurrent infections^2^. The disease associated with CDI results primarily from two homologous large protein toxins, toxin A (TcdA, ~306 kDa) and toxin B (TcdB, ~270 kDa)^5–8^. A subset of *C. difficile* strains also express a third toxin, known as binary toxin CDT^9–11^. TcdA and TcdB belong to a family of large clostridial toxins with similar structures and functions, including TcsL and TcsH in *Paeniclostridium sordellii*, Tcnα in *Clostridium novyi*, and TpeL in *Clostridium perfringens*^8,12,13^.

TcdA and TcdB contain an N-terminal glucosyltransferase domain (GTD), followed by a cysteine protease domain (CPD), an intermingled membrane translocation delivery and receptor-binding domain (DRBD), and a C-terminal domain containing combined repetitive oligopeptides (CROPs)^5–8,13–15^. These toxins target and enter cells via receptor-mediated endocytosis. A reduction in pH within endosomes induces membrane translocation of the GTD and CPD^16,17^. GTD is then separated via autoproteolytic cleavage of the CPD^18^, where GTD targets and inhibits small GTPases by glucosylation of a key residue with UDP-glucose as sugar donor^19,20^. Inhibition of small GTPases disrupts the actin cytoskeleton and leads to morphological changes of intoxicated cells such as cell rounding.

There is growing evidence that TcdB is the major virulence factor and key therapeutic target for CDI in humans^21–24^. TcdA and TcdB differ regarding their receptor-specificity. TcdB has been demonstrated to utilize members of the Wnt receptor frizzled 1/2/7 (FZD1/2/7) and chondroitin sulfate proteoglycan 4 (CSPG4) as receptors, which are recognized through distinct binding sites^25–30^, and are independent from each other^26,31^. In addition, poliovirus receptor-related 3 (PVRL3) and low-density lipoprotein receptor-related protein 1 (LRP1) have been reported as potential receptors^32,33^. TcdA can utilize sulfated glycosaminoglycan and low-density lipoprotein receptor (LDLR) family members to bind and enter cells^34,35^, as well as binding broadly to cell surface carbohydrates via its CROPs domain^36^. Other reported receptors for large clostridial toxins include low-density lipoprotein receptor-related protein 1 (LRP1) for TpeL^37^, semaphorin 6A and 6B (SEMA6A/6B) for TcsL^38,39^, and LDLR/sulfated glycosaminoglycan for Tcnα^40^. The structures of TcdB-FZD and TcdB-CSPG4 complexes have been resolved^27,28^. A well-defined FZD-binding site is located within the middle region of the DRBD domain. Interestingly, a binding site for SEMA6A/6B has been identified at an identical location on the DRBD domain of TcsL^38,39^, which is a toxin sharing ~76% sequence identity with TcdB. The CSPG4-binding site is spatially composed of discontinuous segments scattered across multiple domains of TcdB^28–30^.

Growing numbers of diverse *C. difficile* lineages have been isolated and analyzed, with many expressing TcdB with sequence variations from the standard toxins of the reference strain VPI10463 (designated TcdA1 and TcdB1). Sequence variations could affect toxin functions and antigenicity^41–48^. Two recent studies proposed to group toxin variants into either 8 or 12 subtypes (TcdB1-B12); subtypes exhibit ~3-15% amino acid sequence divergence^49,50^. Furthermore, each unique sequence variant within a subtype can be labeled numerically such as TcdB1.1 etc. ^50^. Compared to TcdB, TcdA showed far less variation (<2%), except for one unique sequence (TcdA7)^50^. A publicly available database containing all known TcdA and TcdB sequences has been established (*DiffBase*, https://diffbase.uwaterloo.ca/)^50^. Sequence comparisons suggest that intragenic recombination (IR) between TcdB variants drives rapid evolution and diversification of TcdB^45,49,50^.

Receptor-recognition dictates the tropism and potency of a toxin. Pathogen-receptor interactions are also major therapeutic targets. Thus, it is critical to understand whether sequence variation in TcdB has led to evolutionary changes in toxin-receptor recognition. Indeed, it has been reported that TcdB2 does not bind to FZD1/2/7 due to residue changes within the FZD-binding site and that TcdB2 shows slightly enhanced binding to CSPG4^31,44,47,51,52^.

Here we collected clinical *C. difficile* isolates that produce representative TcdB variants and systemically investigated their dependency on FZD1/2/7 and CSPG4 receptors, utilizing our FZD1/2/7 knockout (KO) and CSPG4 KO cells^26^. We found that TcdB variants can be divided into two groups: TcdB1/3/5/6/8/9 utilize FZD1/2/7, whereas TcdB2/4/7/10/11/12 do not require FZD1/2/7. We further focused on TcdB4.2, which showed no major reduction in potency on either FZD KO or CSPG4 KO cells compared with wild type (WT) cells, suggesting binding to an alternative receptor. We carried out a genome-wide CRISPR-Cas9 mediated screen using TcdB4.2 and identified tissue factor pathway inhibitor (TFPI) as a receptor. The TFPI-binding site in TcdB4 overlaps with the FZD-binding site in TcdB1. Analyzing all available TcdB sequences further revealed a set of six critical residues that define a TFPI-binding-associated haplotype within this receptor-binding site. Furthermore, frequent IR events around this receptor-binding site have generated diverse TcdB7 and TcdB2 members that can utilize TFPI or FZD1/2/7 as their receptors. Interestingly, TcdB10 has independently evolved the ability to bind TFPI from TcdB4/B7 and showed strong species preference: recognizing TFPI of chicken, to a lesser degree mouse, but not of human, dog, or cattle versions. These findings identify TFPI as a major receptor for TcdB variants, reveal a unique haplotype associated with receptor-binding specificity, and highlight the key role of IR in driving rapid alternation in receptor-specificity for TcdB.

## Results

### Binary variation on FZD-dependency among TcdB subtypes

To systematically investigate functional variation among TcdB subtypes, we collected clinical isolates that express representative TcdB variants (Supplementary Table 1)^53–58^. We either utilized culture supernatants directly as sources of toxins or produced TcdB variants in *Bacillus megaterium* recombinantly. TcdB activity was assessed using the classic cytopathic cell-rounding assay on HeLa cells (Supplementary Fig. 1). Cell-rounding was in all cases prevented by the addition of a polyclonal TcdB antibody, confirming the role of TcdB (Supplementary Fig. 1). Although some isolates also express TcdA and CDT, it is likely that the amount of TcdA and CDT in diluted supernatants is not sufficient to have a major impact on cell rounding under our assay conditions.

TcdB variants are known to cause two types of cell-rounding depending on sequence variations in their GTD domains^20,48,54,59^: TcdB1/2/11 cause an arborizing cytopathic effect (rounded cells with protrusions), whereas TcdB3/4/7 cause a variant cytopathic effect of rounded cells without protrusions and show characteristic clustering. The latter is similar to the cell-rounding caused by TcsL^59^. Our previous sequence comparison indicated that TcdB5/6/9/10/12 contain TcdB1/2/11-like GTDs, whereas TcdB8 has a TcdB3/4/7-like GTD^50^. This prediction is validated in our current analysis: TcdB1/2/5/6/9/10/11/12 induced rounded cells with protrusions and TcdB3/4/7/8 induced TcsL-like phenotype with rounded cells without protrusions (Supplementary Fig. 2a). Therefore, TcdB subtypes showed a binary variation based on their GTD domains.

**Fig. 1:**
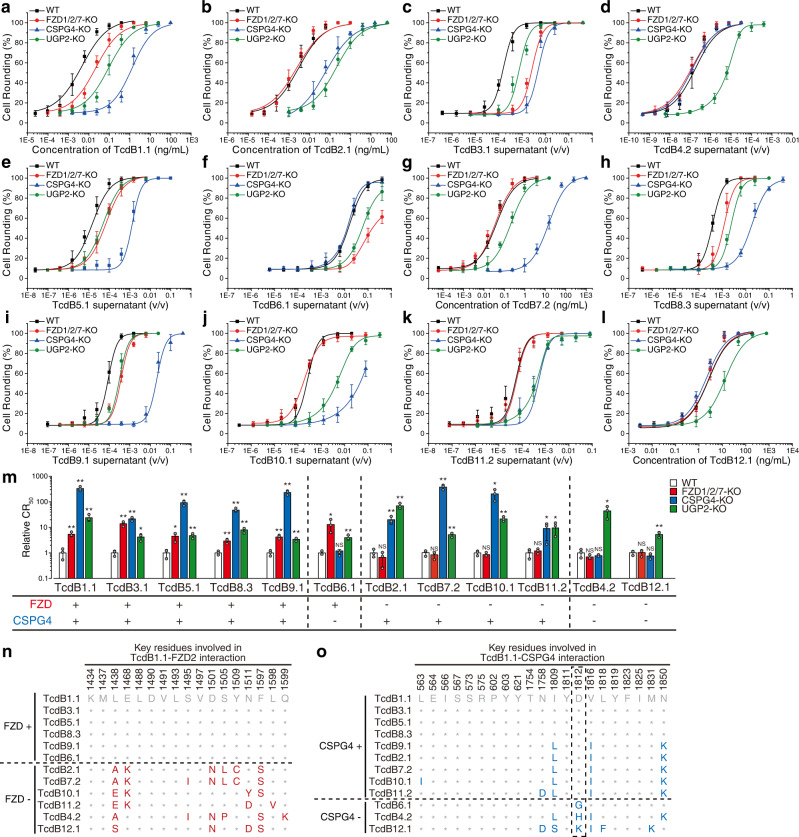
TcdB subtypes show variable dependency on FZD and CSPG4 receptors. HeLa wildtype (WT), stable FZD1/2/7 knockout (FZD1/2/7-KO), CSPG4-KO, and UGP2-KO cells were exposed to either recombinant TcdB1.1 (**a**), TcdB2.1 (**b**), TcdB7.2 (**g**), and TcdB12.1 (**l**), or culture supernatants from native *C. difficile* strains expressing TcdB3.1 (**c**), TcdB4.2 (**d**), TcdB5.1 (**e**), TcdB6.1 (**f**), TcdB8.3 (**h**), TcdB9.1 (**i**), TcdB10.1 (**j**), and TcdB11.2 (**k**) for 24 h. The percentages of round-shaped cells were plotted over toxin concentrations or supernatant dilutions. Error bars indicate mean ± s.d., *N* = 3 (biologically independent experiments). Strain information is listed in Supplementary Table 1. The activity of TcdB in each supernatant has been validated using a polyclonal TcdB antibody as shown in Supplementary Fig. 1. **m** Summary of the receptor preference of TcdB variants. The toxin concentrations inducing 50% of cell rounding (CR_50_) were determined. The relative CR_50_ values in different cell lines were normalized to WT and plotted as a bar-chart. The dependency on FZDs and CSPG4 are noted with plus and minus signs. Error bars indicate mean ± s.d.; *N* = 3 (biologically independent experiments); *, *p* < 0.05; **, *p* < 0.01; NS not significant (Student’s *t*-test, two-sided). **n** A list of residues across tested TcdB subtypes at 17 key positions mediating TcdB1.1-FZD2 interactions. These positions are based on the crystal structure of TcdB-FZD complex (PDB: 6C0B)^27^. **o** A list of residues across tested TcdB subtypes at 21 key positions mediating TcdB1.1-CSPG4 interactions. These positions are based on the cryo-EM structure of TcdB-CSPG4 complex (PDB: 7ML7)^28^. Residue 1812 was highlighted with a dash box. Source data are provided as a Source Data file.

We next analyzed TcdB variants on FZD1/2/7 or CSPG4 KO HeLa cells, in comparison with WT HeLa cells (Fig. 1a–m and Supplementary Fig. 2b). The toxin concentration or supernatant dilution ratio resulting in the rounding of 50% of cells is designated cell-rounding fifty value (CR_50_) for comparing the sensitivity of cells. HeLa cells lacking UDP-glucose pyrophosphorylase 2 (UGP2), which synthesizes UDP-glucose required for TcdB enzymatic activity^60^, were analyzed as a control, which showed reduced sensitivity to all TcdB subtypes (Fig. 1a–m).

There is a clear binary division on FZD1/2/7 KO cells, with reduced sensitivity to TcdB1/3/5/6/8/9 and no change in sensitivity to TcdB2/4/7/10/11/12 compared with WT cells (Fig. 1m). Structure-based alignment of key FZD-binding residues divides TcdB subtypes into two groups, corresponding to their dependency on FZD1/2/7 in cell-rounding assays (Fig. 1n and Supplementary Fig. 3). This binary separation is consistent with our previous analysis classifying DRBD sequences of TcdB variants into two distinct origins^50^. The binary separation on FZD1/2/7-dependency does not match exactly the variations on cell-rounding phenotype caused by the GTD domain. This is because many subtypes are originated from IR events that shuffle GTD and DRBD domains^50^

### Variations at residue 1812 influence CSPG4-dependency

In contrast to FZD1/2/7, CSPG4 is utilized by the majority of TcdB subtypes (Fig. 1a–m). Alignment of key CSPG4-binding residues suggests that residue changes at D1812 in a subset of TcdB members are likely responsible for their reduced dependency on CSPG4. For instance, TcdB6 has only a single residue difference from TcdB1 across key CSPG4-binding residues: D1812 in TcdB1 becomes G1812 in TcdB6 (Fig. 1o and Supplementary Fig. 3). This is consistent with our previous finding that the single D1812G mutation is sufficient to abolish binding of TcdB1 to CSPG4^28^. Alignment of all 206 TcdB sequences revealed that all known members of TcdB6 contain G1812, and thus are expected to be CSPG4-independent (Supplementary Fig. 3).

The TcdB4 strain we utilized here produces TcdB4.2, which contains H1812 (Fig. 1o). H1812 is present in three (out of 24) members of TcdB2, three (out of 6) members of TcdB4, and two (out of 17) members of TcdB7 (Supplementary Fig. 3). To further test the impact of H1812, we produced TcdB2.2 and TcdB7.1 recombinantly in *Bacillus megaterium*. Both contain H1812 and showed no reduction in potency on CSPG4 KO cells (Supplementary Fig. 4). We further tested a culture supernatant containing TcdB7.9, which has D1812, and it showed dependency on CSPG4 in HeLa cells (Supplementary Fig. 4d–g). TcdB12 is the least conserved subtype and showed a relatively low activity on HeLa cells (Fig. 1l). It contains K1812, which could be the reason for its lack of dependency on CSPG4 in HeLa cells.

### CRISPR screening identifies TFPI as a receptor for TcdB4

**Fig. 2:**
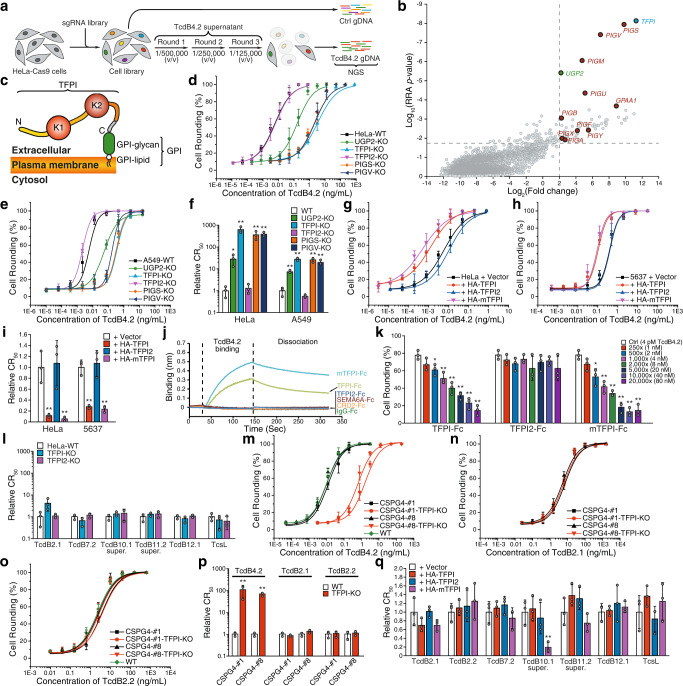
A CRISPR-Cas9 screen identifies TFPI as a receptor for TcdB4. **a** Schematic diagram of the CRISPR-Cas9 screen process. **b** Genes identified by NGS were analyzed with the MAGeCK program^61^ and plotted based on the log_2_ value of fold change of NGS reads and statistical significance (shown as log_10_ value of RRA *p*-value and plotted as the *y*-axis). The genes involved in the GPI (glycosylphosphatidylinositol) biosynthetic pathway are colored red. **c** Schematic diagram of human TFPIβ (TFPI), a GPI-anchored protein with two BPTI/Kunitz protease inhibitor domains (K1 and K2). N, N-termini; C, C-termini. HeLa (**d**) or A549 (**e**) KO cells lacking TFPI, TFPI2 (a homolog of TFPI), PIGS, or PIGV were generated via the CRISPR-Cas9 approach. UGP2-KO cells were also analyzed as a control. Cells were exposed to recombinant TcdB4.2 for 24 h. Their CR_50_ values are normalized to WT and plotted in a bar-chart (**f**). Error bars indicate mean ± s.d.; *N* = 3 (biologically independent experiments); *, *p* < 0.05; **, *p* < 0.01 (Student’s *t*-test, two-sided). **g**–**i** HeLa (**g**) or 5637 (**h**) cells overexpressing triple-HA-tagged TFPI, TFPI2, or mouse TFPI (mTFPI) via lentiviral transduction were exposed to TcdB4.2 for 24 h. The percentages of rounded cells were plotted over toxin concentrations. Their CR_50_ values are normalized to WT and plotted in a bar-chart (**i**). Error bars indicate mean ± s.d.; *N* = 3 (biologically independent experiments); **, *p* < 0.01 (Student’s *t*-test, two-sided). **j** Binding of TcdB4.2 (500 nM) to Fc-tagged TFPI, TFPI2, and mTFPI (immobilized onto capture biosensors) was examined using biolayer interferometry (BLI) assays. Fc-tagged extracellular domains of FZD2 (CRD2), SEMA6A, and IgG were used as controls. Representative sensorgrams from one of three independent experiments are shown. **k** HeLa cells were exposed to either TcdB4.2 alone (4 pM) or TcdB4.2 pre-incubated with Fc-tagged TFPI, TFPI2, or mTFPI at the indicated molar ratios (1:250 ~ 1:20,000) on ice for 1 h. The percentage of cell-rounding at 6 h incubation was plotted. Error bars indicate mean ± s.d.; *N* = 3 (biologically independent experiments); *, *p* < 0.05; **, *p* < 0.01 (Student’s *t*-test, two-sided). **l** HeLa-WT, TFPI-KO, and TFPI2-KO cells were exposed to recombinant TcdB2.1, TcdB7.2, TcdB12.1, TcsL, or culture supernatants of *C. difficile* strains expressing TcdB10.1 or TcdB11.2 for 24 h. The percentages of rounded cells were plotted over toxin concentrations are shown in Supplementary Fig. 8a. Their CR_50_ values were normalized to WT and plotted here. Error bars indicate mean ± s.d.; *N* = 3 (biologically independent experiments). **m**–**p** HeLa-WT, two CSPG4 KO single clones (CSPG4#1 and CSPG4#8), and two CSPG4/TFPI double KO cells (CSPG4#1-TFPI-KO and CSPG4#8-TFPI-KO) were exposed to TcdB4.2 (**m**), TcdB2.1 (**n**), or TcdB2.2 (**o**) for 24 h. The percentages of rounded cells were plotted over toxin concentrations. Their relative CR_50_ values are plotted in a bar-chart (**p**). Error bars indicate mean ± s.d.; *N* = 3 (biologically independent experiments); **, *p* < 0.01 (Student’s *t*-test, two-sided). **q** HeLa cells overexpressing HA-tagged TFPI, TFPI2, or mTFPI via lentiviral transduction were exposed to recombinant TcdB2.1, TcdB2.2, TcdB7.2, TcdB12.1, TcsL, or culture supernatants of *C. difficile* strains expressingTcdB10.1 or TcdB11.2 for 24 h. The percentages of rounded cells were plotted over toxin concentrations and are shown in Supplementary Fig. 8e. Their CR_50_ values are normalized to WT and plotted here. Error bars indicate mean ± s.d.; *N* = 3 (biologically independent experiments); **, *p* < 0.01 (Student’s *t*-test, two-sided). Source data are provided as a Source Data file.

TcdB4.2 showed high potency on HeLa cells, but no reduction on FZD1/2/7 or CSPG4 KO HeLa cells, suggesting the possibility of additional receptors. We thus carried out a genome-wide CRISPR-Cas9-mediated screening on HeLa cells for TcdB4.2 receptors (Fig. 2a). To this end, a genome-wide single guide RNA (sgRNA) library (GeCKO-V2) was transduced into HeLa cells stably expressing Cas9 (Fig. 2a and Supplementary Fig. 5a). Cells were then selected with increasing concentrations of TcdB4.2 supernatant for three rounds (Fig. 2a). Cells that became resistant to TcdB4.2 were harvested and their disrupted genes were identified by next-generation sequencing (NGS) and ranked with MAGeCK (Fig. 2b, Supplementary Fig. 5a, and Supplementary Data 1)^61^.

The top hit is TFPI, a well-known anticoagulation protein limiting tissue factor (TF)-induced coagulation after vascular injury^62,63^. TF initiates the blood coagulation cascade by forming a complex with the activated factor VII (FVIIa) in blood^64^. The TF-FVIIa complex subsequently activates factor X (FXa) and factor IX. TFPI binds to and inhibits TF-FVIIa-FXa complexes through its multiple Kunitz-type protease inhibitory domains. TFPI has two major splicing forms in humans: TFPIα and TFPIβ^62,63^. TFPIα contains three Kunitz-type domains (K1-K3, Supplementary Fig. 5b). It is secreted into the extracellular matrix and may also attach to cell surfaces via its basic carboxyl region. TFPIβ contains a glycosylphosphatidylinositol (GPI) anchor in place of the K3 domain and C-terminal region and is the major form on cell surfaces (Fig. 2c and Supplementary Fig. 5b). Consistently, proteins involved in biosynthesis of the GPI anchor, such as GPI transamidase component PIG-S (PIGS), GPI mannosyltransferase 2 (PIGV), GPI mannosyltransferase 1 (PIGM), phosphatidylinositol glycan anchor biosynthesis class U protein (PIGU), and glycosylphosphatidylinositol anchor attachment 1 protein (GPAA1), were also identified as top hits in our screening (Fig. 2b and Supplementary Fig. 5c).

To validate our screening results, we generated stable KO cell lines lacking TFPI, PIGS, or PIGV in HeLa cells using the CRISPR-Cas9 approach. To exclude cell line dependency, we also generated the same set of KO cells in A549 cells, a human lung cancer cell line that we found to be sensitive to TcdB4.2. KO cells lacking TFPI2, a homolog of TFPIα (Supplementary Fig. 5b), and UGP2 KO cells were analyzed as additional controls. We also purified TcdB4.2 recombinantly using *B. megaterium* for subsequent studies (Supplementary Fig. 4b). HeLa and A549 cells lacking TFPI, PIGS, or PIGV all showed drastically lower susceptibility to TcdB4.2 (Fig. 2d–f and Supplementary Fig. 6a). TFPI2 KO HeLa and A549 cells showed no change in sensitivity. On the other hand, over-expression of either human TFPIβ (TFPI) or a mouse TFPIβ (mTFPI), but not TFPI2, elevated the sensitivity to TcdB4.2 in HeLa cells, and in a human bladder carcinoma cell line 5637 (a cell line that we found to be not sensitive to TcdB4.2) (Fig. 2g–i and Supplementary Fig. 6b).

To assess direct interactions between TcdB4 and TFPI in vitro, we produced human IgG-Fc-tagged TFPI (without the GPI anchor), mTFPI, and TFPI2 using mammalian cells and then carried out biolayer interferometry (BLI) assays with anti-Fc probes. TcdB4.2 showed specific binding to human and mouse TFPI, but not TFPI2 (Fig. 2j). The dissociation constants (*K*_D_) were estimated at ~227 nM for TFPI and ~63 nM for mTFPI (Supplementary Fig. 7a, b, o). TcdB4.2 did not bind to control IgG, the Fc-tagged extracellular domain of FZD2 (CRD2-Fc), or the extracellular domain of SEMA6A (Fig. 2j). Reciprocally, TcdB1.1 and TcsL showed no binding to human or mouse TFPI (Supplementary Fig. 6c, d). Finally, pre-incubation of TcdB4.2 with Fc-tagged human or mouse TFPI reduced toxin-induced cell-rounding on HeLa cells, whereas Fc-tagged TFPI2 showed no effect, confirming that TFPI mediates functional binding and entry of TcdB4 to cells (Fig. 2k and Supplementary Fig. 6e–h).

We next examined members of TcdB variants that do not recognize FZDs (Fig. 1n), including TcdB2.1, 7.2, 10.1, 11.2, and 12.1, by comparing their activity on WT versus TFPI KO cells. We also tested TcsL as an additional control. TcdB7.2, 10.1, 11.2, 12.1, and TcsL showed no change in potency on TFPI KO cells (Fig. 2l and Supplementary Fig. 8a). TcdB2.1 showed a slight reduction in potency on TFPI KO cells but did not reach statistical significance under our assay conditions (Fig. 2l). As TcdB2.1 is dependent on CSPG4 on HeLa cells (Fig. 1b), we carried out three additional studies that minimize the contribution of CSPG4 in order to further clarify the potential role of TFPI for TcdB2: (1) TcdB2.2, which showed no dependency on CSPG4 (Supplementary Fig. 4a), showed no reduction in potency on TFPI KO cells (Supplementary Fig. 8b); (2) a truncated TcdB2.1 (residues 1-1833), which lacks the CROPs and does not bind to CSPG4^26^, showed no reduction in potency on TFPI KO cells (Supplementary Fig. 8c); and (3) we generated two independent CSPG4/TFPI double KO HeLa cell lines. They showed reduction in sensitivity to TcdB4.2 as expected, but no change in sensitivity to TcdB2.1 and TcdB2.2 compared with their parental CSPG4 KO cells (Fig. 2m–p and Supplementary Fig. 8d). Together, these results suggest that TFPI is not a relevant receptor for TcdB2 on HeLa cells.

We also assessed whether over-expression of TFPI increases the sensitivity of cells to TcdB subtypes (Fig. 2q and Supplementary Fig. 8e). Over-expression of TFPI or mTFPI did not increase the sensitivity of HeLa cells to TcdB2.1, 2.2, 7.2, 11.1 and 12.1. To our surprise, over-expression of mTFPI, but not human TFPI, rendered HeLa cells more sensitive to TcdB10.1 (Fig. 2q and Supplementary Fig. 8e). Consistent with this finding, mTFPI-Fc, but not TFPI-Fc protein, reduced the activity of TcdB10.1 on HeLa cells (Supplementary Fig. 9a–c). TFPI-Fc and mTFPI-Fc did not affect the activity of TcdB7.2, 11.2, and 12.1 (Supplementary Fig. 9d–i).

### TFPI-binding site overlaps with FZD-binding site

**Fig. 3:**
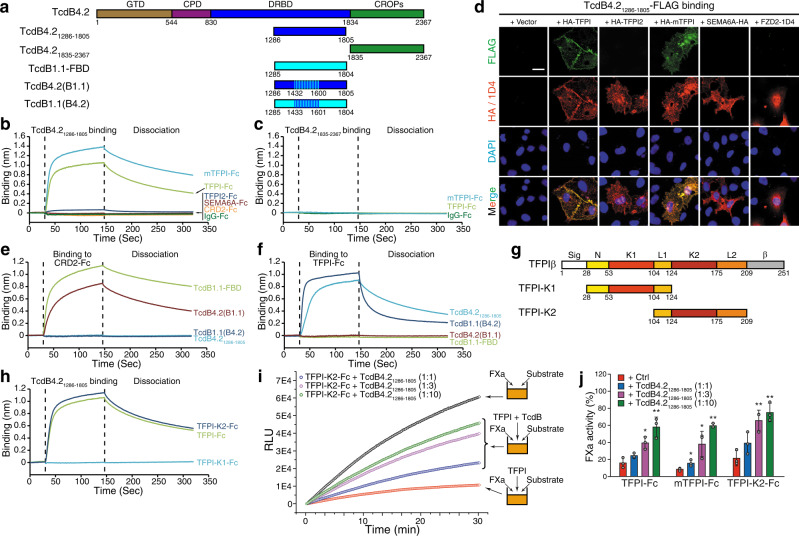
Characterizing TFPI-TcdB4 interactions. **a** Schematic diagrams of TcdB4.2, TcdB4.2_1286–1805_, TcdB4.2_1835–2367_, TcdB1.1-FBD. TcdB4.1(B1.1) and TcdB1.1(B4.2) represent two mutant fragments exchanging 29 residues in the region 1432– 1600 that differ between TcdB4.1 and TcdB1.1. The numbers indicate the position of amino acid residues. GTD, glucosyltransferase domain; CPD, cysteine protease domain; DRBD, delivery/receptor-binding domain; CROPs, combined repetitive oligopeptides. Binding of 500 nM TcdB4.2_1286–__1805_ (**b**) or TcdB4.2_1835–__2367_ (**c**) to Fc-tagged TFPI and mTFPI was examined using BLI assays. Fc-tagged TFPI2, CRD2, SEMA6A, and IgG were used as controls. Representative sensorgrams from one of three independent experiments are shown. **d** HeLa cells transiently transfected with TFPI, TFPI2, mTFPI, SEMA6A, or FZD2 were exposed to FLAG-tagged TcdB4.2_1286–__1805_ (5 µg/mL) on ice for 60 min, washed, fixed, permeabilized, and subjected to immunostaining analysis. Expression of exogenous proteins was confirmed by detecting fused HA or 1D4 tag. Nuclei were labeled with DAPI (blue). Scale bar, 5 µm. Representative images were from one of three independent experiments. Binding of 500 nM TcdB1.1-FBD, TcdB4.2_1286–__1805_, TcdB4.2(B1.1), and TcdB1.1(B4.2) to Fc-tagged CRD2 (**e**) and TFPI (**f**) was examined using BLI assays. Representative sensorgrams from one of three independent experiments are shown. **g** Schematic diagram of TFPIβ, TFPI-K1, and TFPI-K2 fragments. Sig, signal peptide; N, N-terminal domain; K1, BPTI/Kunitz inhibitor domain 1; L1, loop 1; K2, BPTI/Kunitz inhibitor domain 2; L2, loop 2; β, GPI anchor sequence for TFPIβ. **h** Binding of 500 nM TcdB4.2_1286-1805_ to Fc-tagged TFPI, TFPI-K1, and TFPI-K2 was examined using BLI assays. Representative sensorgrams from one of three independent experiments are shown. **i**, **j** TcdB4.2 binding to TFPI-K2 prevents TFPI-K2 binding to its natural ligand coagulation factor Xa (FXa). FXa (0.5 ng/mL) cleaves its fluorescently labeled substrate and generates increasing fluorescent signal (measured as relative light unit, RLU, *y*-axis) over time (*x*-axis). FXa’s enzymatic activity was inhibited by TFPI-K2 (**i**, 7.5 ng/mL). The inhibitory effect of TFPI was blocked by adding TcdB4.2_1286–__1805_ in a dose-dependent manner (1:1, 1:3, or 1:10 molar ratio). Representative curve from one of three independent experiments is shown. FXa activity was quantified by measuring the slope of RLU curves (1–10 min) and plotted as a bar-chart (**j**). The same experiments were also carried out for TFPI-Fc and mTFPI-Fc, with their curves shown in Supplementary Fig. 10c, d and quantification in (**j**). Error bars indicate mean ± s.d.; *N* = 3; *, *p* < 0.05; **, *p* < 0.01 (Student’s *t*-test, two-sided). Source data are provided as a Source Data file.

To further characterize TcdB4-TFPI interactions, we next generated a TcdB4.2 fragment (residue 1286–1805, Fig. 3a), based on a TcdB1 fragment previously shown to be well-folded and to contain the FZD-binding site (known as TcdB-FBD)^27^. This fragment (TcdB4_1286–__1805_) binds to both human and mouse TFPI (*K*_D_ ~ 50 nM for TFPI and ~ 24 nM for mTFPI) (Fig. 3b and Supplementary Fig. 7c, d, o), with no binding to Fc-tagged TFPI2, CRD2, SEMA6A, or IgG (Fig. 3b). As a control, the CROPs domain of TcdB4.2 (residues 1835–2367) showed no binding to human or mouse TFPI (Fig. 3a, c). Furthermore, TcdB4_1286–1805_ was able to bind to the surface of HeLa cells transfected with human or mouse TFPI, but not TFPI2, SEMA6A, or FZD2 (Fig. 3d).

We next switched 29 residues within the FZD-binding interface region (between 1433 and 1601) that are different between TcdB1.1 and TcdB4.2, creating a TcdB1 fragment containing TcdB4-derived residues (designated TcdB1.1(B4.2)) and a TcdB4 fragment containing TcdB1-derived residues (designated TcdB4.2(B1.1)) (Fig. 3a). TcdB4.2(B1.1) binds to FZD-CRD2, but not TFPI, while TcdB1.1(B4.2) binds to human and mouse TFPI, but not FZD-CRD2 (Fig. 3e, f and Supplementary Fig. 10a). These results suggest that the TFPI-binding site in TcdB4 overlaps with the FZD-binding site in TcdB1 and that differences in residues dictate specificity towards FZDs or TFPI.

### TcdB4 recognizes the K2 domain of TFPI

To map the TcdB binding site on TFPI, we generated fragments of TFPI containing either its K1 (TFPI-K1) or K2 domain (TFPI-K2, Fig. 3g). Both full-length TcdB4 and TcdB4_1286–__1805_ bound to TFPI-K2, but not to TFPI-K1 (Fig. 3h and Supplementary Fig. 10b). As TFPI-K2 is also the domain responsible for binding to FXa^65^, we further evaluated whether binding of TcdB4 is capable of blocking TFPI-FXa interactions, utilizing an in vitro FXa inhibition assay^66^. Cleavage of a substrate by FXa generates fluorescence signals and adding TFPI-K2 can bind to FXa and sequester FXa’s activity. Pre-mixing of TcdB4_1286–__1805_ with TFPI-K2 resulted in higher FXa activity in a dose-dependent manner, suggesting that binding of TcdB4_1286–__1805_ prevents TFPI-K2 from sequestering FXa (Fig. 3i, j). TcdB4_1286–__1805_ also reduced the inhibition from TFPI and mTFPI in this assay (Fig. 3j and Supplementary Fig. 10c, d). Thus, TcdB4 recognizes the K2 domain and blocks the anticoagulation function of TFPI.

### TFPI is a receptor for TcdB4 in intestinal organoids and lung tissues

To next investigate the role of TFPI in vivo, we initially carried out cecum injection assays as previously described^28^. However, histological analysis revealed little damage to cecal tissue with as high as 200 µg of TcdB4.2, whereas TcdB1.1 at 6 µg already caused severe damage (Supplementary Fig. 11a). To examine whether TcdB4.2 is deactivated within the mouse cecum, we harvested cecal contents 6 hours after injection of the same amount of TcdB1.1 or TcdB4.2 (8 µg). These two toxins showed similar potency on inducing cell-rounding of HeLa cells before injection, but recovered TcdB4 samples showed several magnitude lower toxicities compared with TcdB1.1 on HeLa cells (Supplementary Fig. 11b), suggesting that purified TcdB4.2 is deactivated within the mouse cecum. We then carried out limited proteolysis analysis by treating TcdB1.1 and TcdB4.2 with trypsin. TcdB1.1 is fairly resistant to trypsin under both pH5.8 and pH7.8 conditions, whereas TcdB4.2 is easily degraded under the same assay conditions (Supplementary Fig. 11c, d). These findings suggest that purified TcdB4.2 may have stability issues within the cecum, which is consistent with a previous report that injection of purified TcdB4 alone did not induce intestinal damage in a rabbit ileal loop model^67^. We note that this previous study utilized TcdB4.1, which shares 99% amino acid sequence identity with TcdB4.2.

**Fig. 4:**
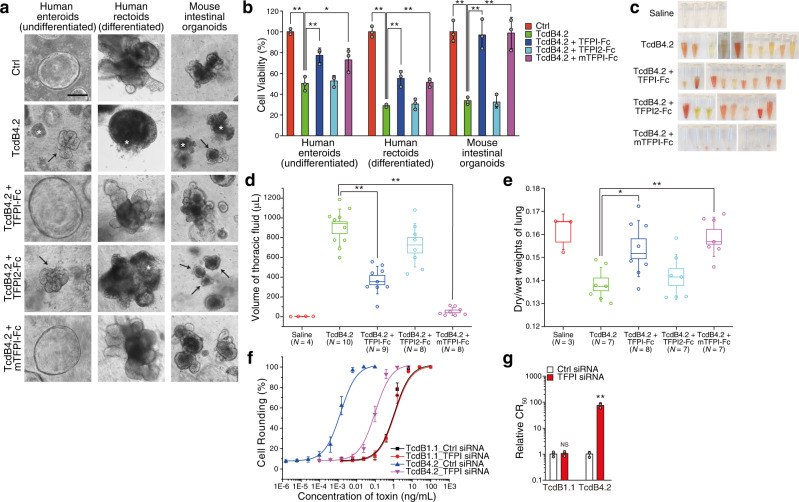
TFPI is a receptor for TcdB4.2 in intestinal organoids and lung tissues. **a** Cultured undifferentiated human enteroids (in growth medium), differentiated human rectoids (in differentiation medium), and mouse intestinal organoids were exposed to either TcdB4.2 alone (10 pM) or TcdB4.2 pre-incubated with Fc-tagged TFPI, TFPI2, or mTFPI (100 nM) for 8 h. PBS was used as control (Ctrl). Stars indicate the dissociated organoids with released luminal contents; arrows indicate shrunken organoids; scale bar, 50 µm. Representative images are from one of three independent experiments. **b** Experiments were carried out as described in panel (**a**). After exposure to toxin for 3 days, cell viability was measured using the MTT assay and plotted as a bar-chart. Error bars indicate ± s.d.; *N* = 3 (biologically independent experiments); *, *p* < 0.05; **, *p* < 0.01 (Student’s *t*-test, two-sided). **c**, **d** Accumulation of fluid in the thoracic cavity occurred within 15 h after intraperitoneal injection of TcdB4.2 into mice (50 ng per 25 g bodyweight). Injection of TcdB4.2 pre-incubated with Fc-tagged TFPI or mTFPI at 1:2000 molar ratios showed less fluid accumulation. Co-injection of TcdB4.2 with Fc-tagged TFPI2 at 1:2000 molar ratio did not affect fluid accumulation. Injection of saline was included as a control. The range of boxes indicates ± s.e.m.; whiskers indicate ± s.d.; percentiles indicate median; **, *p* < 0.01 (Student’s *t*-test, two-sided). **e** Experiments were carried out as described in panel (**c**) and the edema in lung tissues was evaluated by calculating dry-to-wet weight ratios. TcdB4.2 reduced dry-to-wet weight ratio of lung tissue more than in the saline group. Co-injection of TcdB4.2 with TFPI-Fc or mTFPI-Fc prevented this reduction, whereas co-injection with TFPI2-Fc showed no protection from TcdB4.2. The range of boxes indicates ± s.e.m.; whiskers indicate ± s.d.; percentiles indicate median; *, *p* < 0.05; **, *p* < 0.01 (Student’s *t*-test, two-sided). **f**, **g** The sensitivity of HUVECs transfected with siRNAs targeting TFPI to TcdB1.1 or TcdB4.2 was analyzed using the 24 h cell-rounding assay. HUVECs transfected with non-targeting scrambled siRNAs served as a control. Dose-response curves are plotted in (**f**), and their relative CR_50_ are plotted in a bar chart (**g**). Error bars indicate ± s.d.; *N* = 3 (biologically independent experiments); **, *p* < 0.01; NS not significant (Student’s *t*-test, two-sided). Source data are provided as a Source Data file.

We then utilized human and mouse intestinal organoid models as an alternative approach. Intestinal organoids are derived from intestinal stem cells and form a single layer of intestinal epithelial cells imbedded in extracellular matrix. Analysis of undifferentiated human enteroids (derived from small intestine), differentiated human rectoids (a proxy for colonoids)^68^, and mouse intestinal organoids revealed each was susceptible to TcdB4.2, based on induced shrinkage, structural disruption, and eventual organoid death quantified by MTT assays (Fig. 4a, b and Supplementary Fig. 11e, f). Pre-incubation of TcdB4.2 with Fc-tagged TFPI or mTFPI protected both human and mouse organoids from TcdB4.2, whereas Fc-tagged TFPI2 showed no protection (Fig. 4a, b and Supplementary Fig. 11e, f). These findings demonstrate that TFPI is a receptor for TcdB4 on intestinal epithelial cells.

To further explore potential differences between TcdB1.1 and TcdB4.2, we next utilized a systemic toxicity mouse model by intraperitoneal (IP) injection of TcdB. Damage to lung tissues is one of the notable pathogenic effects observed with TcdB4.2 in this assay, with severe accumulation of thoracic fluid and edema (Fig. 4c–e), whereas the same dose of TcdB1.1 caused less damage to lung tissues under the same assay conditions (Supplementary Fig. 11g–i). Co-injection of Fc-tagged TFPI or mTFPI with TcdB4.2 (2000:1 ratio) reduced thoracic fluid and dry/wet weights of lung tissues (Fig. 4c–e and Supplementary Fig. 11j). Fc-tagged TFPI2 showed no effect. TFPI is expressed in lung endothelial cells (Supplementary Fig. 11k)^64^, which may contribute to the phenotypes observed on lung tissue in vivo. To further confirm the role of TFPI in human endothelial cells, we utilized primary human umbilical vein endothelial cells (HUVECs) as a model. Knocking down TFPI expression in HUVECs using the RNA interference approach reduced the susceptibility of these cells to TcdB4.2, whereas their susceptibility to TcdB1.1 was not altered (Fig. 4f, g and Supplementary Fig. 11l, m).

### Sequence comparison reveals the B4/B7-haplotype and intragenic recombination

**Fig. 5:**
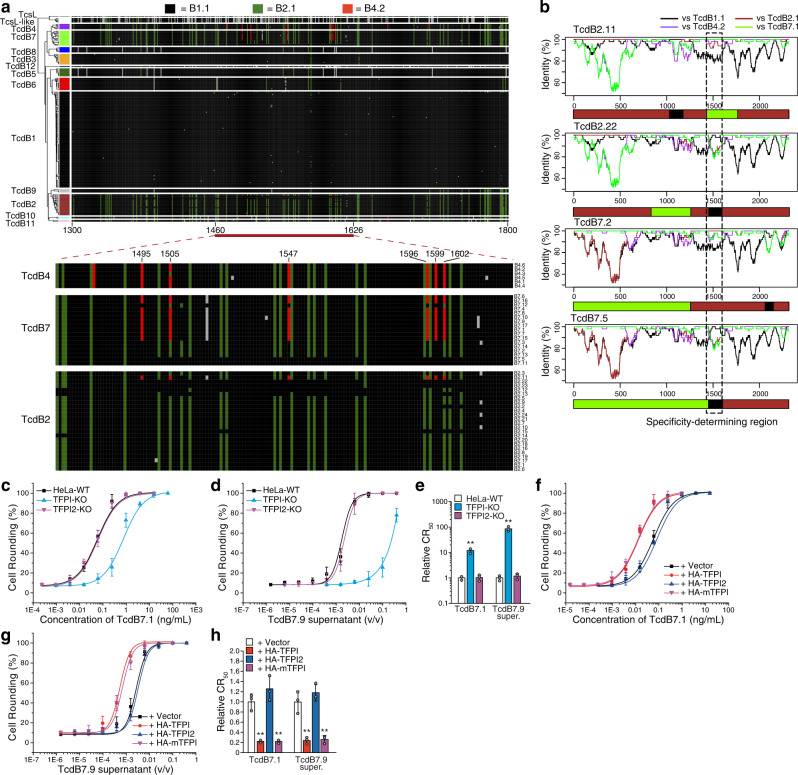
Sequence comparison of TcdB variants reveals B4/B7-haplotype and IR events. **a** Amino acids across all 206 known TcdB sequences and 6 TcsL sequences were aligned and visualized using a haplotype coloring algorithm we recently developed^50^, showing variation patterns across TcdB members. The first sequence (TcdB1.1) is assigned black color, and all other sequences colored black if they share the same residues. Unique residues in the second sequence (TcdB2.1) are colored green, followed by unique residues in the third sequence (TcdB4.2) colored red. The region 1460 to 1626 is enlarged for TcdB2/4/7 members. A unique B4/B7-haplotype can be visualized with residues in red color, with their position marked. Residue A1518 is unique in TcdB7 members and is colored gray. **b** The sequences of TcdB2.11, TcdB2.22, TcdB7.2, and TcdB7.5 were analyzed using a sliding window comparison with TcdB1.1, 2.1, 4.2, and 7.1, revealing their recombination patterns. Below each sliding window plot is a graphical summary depicting the recombination pattern. The location surrounding the TFPI/FZD-binding site (specificity-determining region) is marked. HeLa-WT, TFPI-KO, and TFPI2-KO cells were exposed to recombinant TcdB7.1 (**c**) or the culture supernatant of *C. difficile* strain expressing TcdB7.9 (**d**) for 24 h. The percentages of round-shaped cells were plotted over toxin or supernatant dilutions. Their CR_50_ values are normalized to WT and plotted in a bar-chart (**e**). Error bars indicate mean ± s.d.; *N* = 3 (biologically independent experiments); **, *p* < 0.01 (Student’s *t*-test, two-sided). HeLa cells overexpressing HA-tagged TFPI, TFPI2, or mTFPI via lentiviral transduction were exposed to recombinant TcdB7.1 (**f**) or the culture supernatant of *C. difficile* strain expressing TcdB7.9 (**g**) for 24 h. The percentages of round-shaped cells were plotted over toxin or supernatant dilutions. Their CR_50_ values were normalized to WT and plotted in a bar-chart (**h**). Error bars indicate mean ± s.d.; *N* = 3 (biologically independent experiments); **, *p* < 0.01 (Student’s *t*-test, two-sided). Source data are provided as a Source Data file.

To understand the determinants of FZD versus TFPI receptor binding by TcdB, we next compared the region 1300–1800 of all 206 known TcdB and six related TcsL family protein sequences. The patterns of amino acid variation were visualized using a haplotype coloring algorithm that we recently developed^50^. We first colored residues black if they matched the standard TcdB1.1, green if they matched TcdB2.1, and then red if they matched TcdB4.2 (Fig. 5a). TcdB2/4/7/10/11, which do not utilize FZDs as receptors, share a general similarity across this region and possess a substitution profile that is distinct from that of TcdB1.1 (Fig. 5a).

We further enlarged the region 1460–1626 for TcdB2, 4, and 7 (Fig. 5a). This procedure highlights a set of specific substitutions shared between TcdB4 and TcdB7 (residues 1495/1505/1547/1596/1599/1602, in red) that are missing in most TcdB2 and all other subtypes (Fig. 5a). These six substitutions form a unique haplotype (defined as B4/B7-haplotype) that is conserved across all six TcdB4 members and linked to an additional substitution (V1516A) in TcdB7 members. Interestingly, TcdB7 members are highly variable within this region: ten sequences contain this haplotype and seven do not, but instead contain sequences either identical to TcdB2.1 (green) or TcdB1.1 (black). In addition, one member of TcdB2, TcdB2.11, contains this B4/B7-haplotype (Fig. 5a). As TcdB2.11 contains a V1516A substitution, its haplotype most likely originates from a member of TcdB7.

The pattern of sequence variations within TcdB7 and TcdB2 members strongly suggests IR events. A sliding window analysis confirmed that TcdB2.11 has acquired two recombination segments—one from TcdB1 near position 1000 and a second from TcdB7 including the B4/B7-haplotype region (Fig. 5b). TcdB2.22, TcdB2.23, TcdB7.5, and TcdB7.11 have acquired segments similar to TcdB1 around the FZD-binding site (Fig. 5b). TcdB7.2 has acquired a segment similar to TcdB2.1 around the B4/B7-haplotype region (Fig. 5b).

### TcdB2.11 and a subset of TcdB7 recognize TFPI

Both TcdB2.1 and TcdB7.2 lack the B4/B7-haplotype. Similar to TcdB2.1, TcdB7.2 showed no reduction in potency on CSPG4/TFPI double KO cells compared with the parental CSPG4 KO cells (Supplementary Fig. 12a), confirming that TcdB7.2 does not utilize TFPI as its receptor. These findings suggest that having the B4/B7-haplotype is critical for recognizing TFPI. To validate this hypothesis, we examined the sensitivity of WT versus TFPI KO HeLa cells to purified TcdB7.1 and culture supernatant of TcdB7.9: both toxins contain the B4/B7-haplotype but vary on CSPG4-dependency (Fig. 5a and Supplementary Fig. 4). Both toxins showed reduced potency on TFPI KO HeLa cells (Fig. 5c–e). Furthermore, over-expression of human and mouse TFPI enhanced the sensitivity of HeLa cells to TcdB7.1 and TcdB7.9 (Fig. 5f–h), and Fc-tagged TFPI and mTFPI reduced toxicity of TcdB7.1 and TcdB7.9, on HeLa cells (Supplementary Fig. 12b–g). These findings demonstrate that TFPI is a receptor for TcdB7.1 and TcdB7.9.

To further validate that IR events switch receptor-binding specificity, we generated fragments of TcdB2.1, 2.11, 7.2, and 7.5 corresponding to TcdB4_1286–__1805_. The fragment of TcdB2.11 represents the ones containing the B4/B7-haplotype and it is identical to TcdB7.1 and 7.9 within the region 1460–1626 (Fig. 5a). The fragment of TcdB7.5 represents the ones containing the potential FZD-binding site and its sequence is identical to TcdB2.22, 2.23, and 7.11 within the region 1460–1626 (Fig. 5a). In addition, we also generated similar fragments of TcdB10.1, 11.2, and 12.1.

**Fig. 6:**
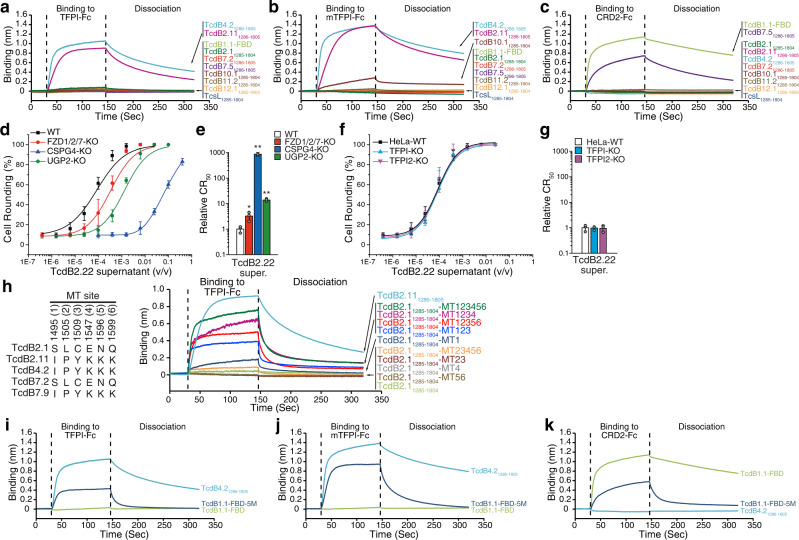
IR generates receptor-switching in TcdB. Binding of 500 nM TcdB1.1-FBD, TcdB2.1_1285–__1804_, TcdB2.11_1286–__1805_, TcdB4.2_1286–__1805_, TcdB7.2_1286–__1805_, TcdB7.5_1286–__1805_, TcdB10.1_1285–__1804_, TcdB11.2_1285–__1804_, TcdB12.1_1285–__1804_, and TcsL_1285–__1804_ to Fc-tagged TFPI (**a**), mTFPI (**b**), or CRD2 (**c**) was examined using BLI assays. Representative sensorgrams from one of three independent experiments are shown. **d**, **e** HeLa-WT, FZD1/2/7-KO, CSPG4-KO, and UGP2-KO cells were exposed to the culture supernatant of a *C. difficile* strain expressing TcdB2.22 for 24 h. The percentages of round-shaped cells were plotted over supernatant dilution (**d**). The relative CR_50_ values in different cell lines were normalized to the WT and plotted as a bar-chart (**e**). Error bars indicate mean ± s.d.; *N* = 3 (biologically independent experiments); *, *p* < 0.05; **, *p* < 0.01 (Student’s *t*-test, two-sided). **f**, **g** HeLa-WT, TFPI-KO, and TFPI2-KO cells were exposed to the culture supernatant of a *C. difficile* strain expressing TcdB2.22 for 24 h. The percentages of round-shaped cells were plotted over supernatant dilutions (**f**). The relative CR50 values in different cell lines were normalized to the WT and plotted as bar-chart (**g**). Error bars indicate mean ± s.d.; *N* = 3 (biologically independent experiments). **h** There are six residues that are different between TFPI-binding TcdB4.2, 2.11, 7.9 versus TcdB2.1 and 7.2 (1495, 1505, 1509, 1547, 1596, and 1599, marked as MT site 1–6). Mutagenesis studies were performed to replace the indicated residues on TcdB2.1_1285-1804_ with the corresponding residues found in TcdB4.2. The binding of the indicated mutant proteins (500 nM) to immobilized TFPI-Fc was analyzed using BLI assays. TcdB2.11_1286–__1805_ and TcdB2.1_1285–__1804_ were analyzed as controls. Representative sensorgrams from one of three independent experiments are shown. **i**–**k** A TcdB1.1-FBD-5M mutant proteins were generated by replacing five residues in TcdB1.1 with the corresponding residues found in TcdB4.2 (positions 1495, 1505, 1547, 1596, and 1599). The binding of this mutant protein to immobilized TFPI-Fc (**i**), mTFPI-Fc (**j**), and CRD2 (**k**) was analyzed using BLI assays. TcdB1.1-FBD and TcdB4.2_1285–__1804_ were analyzed as controls. Source data are provided as a Source Data file.

We then analyzed binding of these fragments to TFPI, mTFPI, and FZD-CRD2 using BLI assays. TcdB2.11 fragment showed robust binding to TFPI and mTFPI, similar to TcdB4_1286–__1805_ (Fig. 6a, b), confirming that TcdB2.11 (and TcdB7 members with the B4/B7-haplotype) can bind to TFPI directly as a receptor. Fragments from TcdB2.1, 7.2, 11.2, and 12.1 showed no binding to TFPI or mTFPI under our assay conditions (Fig. 6a, b), consistent with our findings that these toxins do not utilize TFPI as a receptor in HeLa cells (Fig. 2l). TcdB10.1 fragment showed binding to mTFPI, but not human TFPI (Fig. 6a, b), suggesting that TcdB10.1 may selectively recognize mTFPI. This is consistent with our findings that over-expression of mTFPI, but not human TFPI, increased the sensitivity of HeLa cells to TcdB10.1 (Fig. 2q) and Fc-tagged mTFPI can reduce activity of TcdB10.1 on HeLa cells (Supplementary Fig. 9a–c).

### TcdB7.5/7.11/2.22/2.23 recognize FZD1/2/7

TcdB7.5_1286–__1805_ fragment showed no binding to TFPI or mTFPI as expected (Fig. 6a, b). Instead, it showed robust binding to FZD-CRD2, similar to TcdB1-FBD (Fig. 6c), suggesting that TcdB7.5, 7.11, 2.22, and 2.23 recognize FZD1/2/7 as their receptors. We further examined the culture supernatant from a clinical isolate that produces TcdB2.22. As expected, it showed reduced potency on FZD1/2/7 KO HeLa cells and CSPG4 KO cells, but not change on TFPI KO cells (Fig. 6d–g), confirming that TcdB2.22 utilizes FZD1/2/7 but not TFPI as its receptors.

### Introducing B4/B7-haplotype into TcdB2.1 enables TFPI binding

Within the region mapped for TFPI binding (residue 1430–1600), there are only five residues present in the B4/B7-haplotype that are not found in TcdB2.1 (Figs. 5a and 6h). We also included residue 1509, as TcdB2.1 has a unique cysteine at this position (TcdB4/7 share a tyrosine at this position with TcdB1). We carried out mutagenesis studies by systemically replacing these six residues in TcdB2.1_1286–__1805_ with the corresponding residues in the B4/B7-haplotype. Replacing all six residues resulted in strong binding to TFPI as expected (Fig. 6h). In fact, replacing just the first three residues (S1495I/L1505P/C1509Y) already resulted in modest binding to TFPI (Fig. 6h). Adding the other three residues further increased binding (Fig. 6h). Interestingly, mutating all five residues except S1495 elicited little binding, even less than that recorded for a mutant containing only S1495I (Fig. 6h), suggesting that the S1495I substitution was critical for gaining TFPI-binding capability within the TcdB2.1 background.

### Introducing B4/B7-haplotype residues into TcdB1.1 enables dual binding to TFPI and FZDs

We next investigated whether the residues differentiating the B4/B7-haplotype from TcdB2.1 are sufficient to mediate binding to TFPI in a TcdB1.1 background. Five residue changes were introduced between 1430-1600 to generate a mutated TcdB1-FBD (designated TcdB1.1-FBD-5M, positions 1495/1505/1547/1596/1599), which can now bind to TFPI and mTFPI, although with ~10-fold reduction in binding affinity compared with TcdB4_1286-1805_ (Fig. 6i, j and Supplementary Fig. 7p–s). Surprisingly, TcdB1.1-FBD-5M retained binding to FZD-CRD2, although with reduced binding affinity (Fig. 6k and Supplementary Fig. 7r, s). It is remarkable that recognition of a completely new receptor can be achieved by substituting only five residues within this interface, and an “evolutionary intermediate” state that recognizes two unrelated receptors can be achieved.

### TcdB10 showed species selectivity on TFPI recognition

**Fig. 7:**
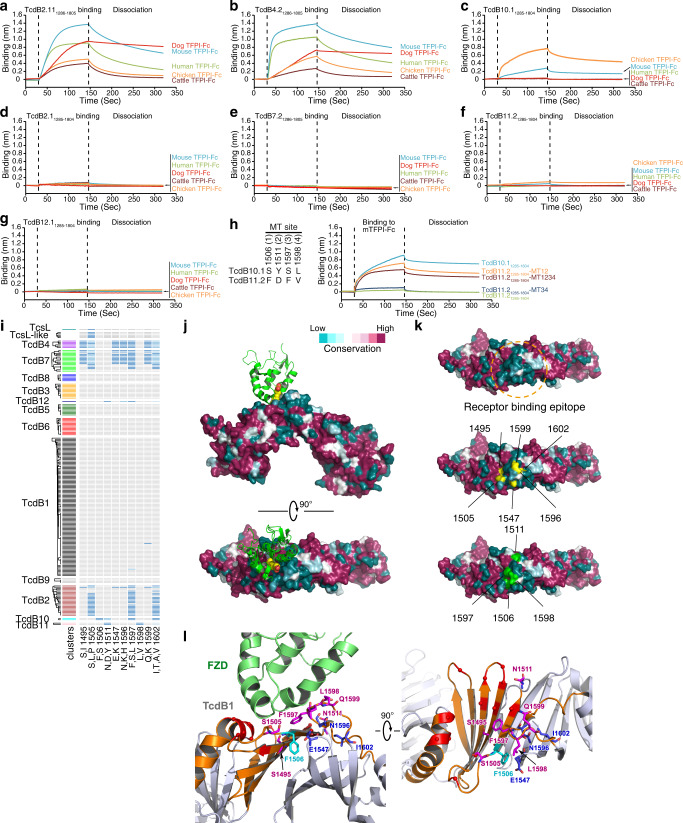
An adaptable receptor-binding interface underlies TcdB diversification. Binding of 500 nM TcdB2.11_1286–__1805_ (**a**), TcdB4.2_1286–__1805_ (**b**), TcdB10.1_1285–__1804_ (**c**), TcdB2.1_1285–__1804_ (**d**), TcdB7.2_1286–__1805_ (**e**), TcdB11.2_1285–__1804_ (**f**), and TcdB12.1_1285–__1804_ (**g**) to Fc-tagged TFPI from the indicated animal species was examined using BLI assays. Representative sensorgrams from one of two independent experiments are shown. **h** There are four different residues between TcdB10.1_1431–__1600_ and TcdB11.2_1431–__1600_ (1506, 1511, 1597, and 1598, marked as MT site 1–4). TcdB11.2_1285–__1804_ mutant fragments were generated by replacing the residues at the indicated positions with the corresponding residues in TcdB10.1. Binding of 2000 nM TcdB10.1_1285-1804_, TcdB11.2_1285–__1804_, and TcdB11.2_1285–__1804_ mutant fragments to Fc-tagged mTFPI was examined using BLI assays. Representative sensorgrams from one of three independent experiments are shown. **i** Plot of amino acid variation for key residues (B4/B7 haplotype and B10-specific substitutions) associated with gain of TFPI-binding in TcdB. A total of 212 sequences were clustered based on sequence identity into 12 distinct subtypes including two TcsL-related groups. A tree depicting these clusters is shown on the left, and on the right the associated residues are shown with gray indicating identity to the TcdB1.1 reference sequence. Variant residues from B1.1 are colored in shades of blue. **j** Surface conservation and variation of TcdB whereas the residues are colored according to their conservation across 206 distinct TcdB sequences. Structure of TcdB1-FZD-CRD2 complex (PDB: 6C0B) was used for the surface presentation. CRD2 and the CDR-bound fatty acid (palmitoleic acid lipid, PAM) were shown as cartoon and spheres and colored as green and yellow, respectively. **k** The sequence diversified region that converged together at the FZD-binding site (receptor binding epitope) on TcdB1 were highlighted by a yellow dashed circle (upper panel). Six residues forming the B4/B7-haplotype were highlighted in yellow (middle panel) and four residues that are different TcdB10 and TcdB11 were highlighted in green (lower panel). **l** Structural location of key residues associated with gain of TFPI-binding is mapped on the structure of the TcdB1-FZD complex (PDB ID 6C0B). The main FZD/TFPI-binding interface on TcdB1 that is composed of a α helix, a β sheet, and a long loop is colored orange, while the rest of the toxin is colored gray. B4/B7-haplotype and B10-specific substitutions are shown as blue and cyan sticks, respectively, while those overlapping with the FZD-binding residues are colored in pink. The rest of the FZD-binding residues on TcdB1 are colored red with their Cα atoms shown as small spheres and their side chains omitted for clarity.

Our findings on TcdB10 suggest that some TcdB variants might adapt toward certain animal hosts. We thus produced additional TFPI-Fc proteins representing major farm and domestic animals including chicken, dog, and cattle, and systemically tested binding of the 1286-1805 fragments of TcdB2.1, 4.2, 2.11, 7.2, 10.1, 11.2, and 12.1. BLI analysis revealed that TcdB2.11 and 4.2 are capable of binding to all the TFPIs tested, with mouse, dog, and human versions being strong binders (Fig. 7a, b and Supplementary Fig. 7c–l, o). TcdB10 showed strong binding to chicken TFPI, higher than its binding to mTFPI, and no binding to human, dog, or cattle TFPI (Fig. 7c and Supplementary Fig. 7m–o). The fragments of TcdB2.1, 7.2, 11.2, and 12.1 showed no binding to any of the TFPIs tested here (Fig. 7d–g). These findings suggest that TcdB2.11/4.2 may act on a broad range of species and are well adapted for human receptors, whereas TcdB10 might be optimized toward certain non-human host species.

### TcdB10 evolved its TFPI binding capability independently

Sequence analysis showed that the B4/B7-haplotype is not shared in TcdB10 (Fig. 5a). TcdB10 is closely related to TcdB11 within the region 1430-1600, with only four residue differences (Fig. 7h, i). We thus carried out mutagenesis studies by replacing residues in TcdB11 with the corresponding residues in TcdB10. Changing just two residues (F1506S/D1511Y) created a mutant TcdB11 fragment that can bind to mTFPI at levels comparable to mutating all four residues (Fig. 7h). Mutating the other two residues (F1597S/V1598L) only resulted in a low level of mTFPI binding (Fig. 7h). Thus, the TcdB10-specific substitutions F1506S and D1511Y are important in TcdB10 for mTFPI binding. As the B4/B7 haplotype contains F1506 and N1511, and TcdB10 contains S1495 (Fig. 7i), this indicates that TcdB10 and TcdB4/B7 have evolved TFPI-binding capability independently.

### An adaptable receptor-binding interface underlies TcdB diversification

We next analyzed the surface amino acid conservation across all 206 TcdB variants in TcdB-FBD region, based on the crystal structure of TcdB1 in complex with FZD-CRD2 (PDB 6C0B)^27^. This analysis showed that the FZD-binding site is more variable than the other regions (Fig. 7j, k). All six residues forming the B4/B7-haplotype and the four residues different between TcdB10 and TcdB11 are clustered on the surface of this receptor-binding site (Fig. 7k). We note that SEMA6 also binds at the identical location on TcsL (Supplementary Fig. 13a).

The FZD-binding site in TcdB1 is composed of three adjacent parts: an α-helix on one side (residues L1433-Y1449), a β-sheet (residues N1459-S1510) in the middle, and a long loop (residues M1588-I1602) on the other side (Fig. 7l). Among the six B4/B7-haplotype residues and four TcdB10-B11 differentiating residues, four (1495, 1505, 1506, and 1511) are in the β-sheet region, and five residues (1596, 1597, 1598, 1599, and 1602) are located on the long loop (Fig. 7l). Residues at six of these positions (1495/1505/1511/1597/1598/1599) in TcdB1 make direct contact with FZD, and four equivalent positions (1495/1505/1597/1599) in TcsL make direct contact with SEMA6 (Fig. 7l and Supplementary Fig. 13b). The overall stability of the β-sheet (residues N1459-S1510) at this site is maintained by lateral backbone hydrogen bonds between β-strands, which leaves their side chains great freedom to be changed. At the same time, residues on the neighboring long loop (residues M1588-I1602) allow diverse residual variation to occur without affecting the local structure in TcdB. These findings suggest that the FZD/TFPI-binding site possesses a highly adaptable interface that can be modulated through only a few residue changes to alter specific protein-protein interactions.

## Discussion

A growing number of TcdB sequence variations have been identified in recent years^45,49,50^. Considering that all TcdB subtypes share high sequence identity (>85%)^50^, it is a surprise that TcdB4, a subset of TcdB7, a member of TcdB2, as well as TcdB10, have evolved the ability to recognize another receptor, TFPI, which is evolutionarily and structurally unrelated to the known toxin receptor FZDs. This receptor switching is mediated by IR around the receptor-binding segment, demonstrating the power of IR in rapidly altering the functional specificity in pathogen evolution. Because of frequent IR, distinct receptor-specificity exists even within the same subtype: TcdB2 has one member (TcdB2.11) that recognizes TFPI and two members (TcdB2.22 and 2.23) that recognize FZD1/2/7. The ability for toxin subtypes with such a high overall sequence identity to adapt to distinct receptors is remarkable, for instance, TcdB7.1 shares 98% identity with TcdB7.5, yet one recognizes TFPI and the other recognizes FZDs as receptors.

The selective pressure that drives receptor switching and the implication of receptor switching on pathogenesis remains to be established. Previous analysis showed that *C. difficile* strains producing TcdB2/4/7 are classified as Clade 2^50,69,70^. Interestingly, TcdB variants containing the B4/B7 TFPI-binding haplotype (e.g.,: TcdB4.1, 4.2, 7.1, 7.4) and ones that do not bind TFPI (e.g.,: TcdB7.2 and 7.3) are found in closely related strains that cluster near one another in a previously constructed genome-wide phylogeny of *C. difficile* strains available in the NCBI database (Supplementary Fig. 14a)^50^. These genomes occur within several sub-lineages of Clade 2 apart from the major sub-lineage containing most Clade 2 strains. The close phylogenetic proximity of these genomes is consistent with the idea of TcdB recombination occurring within closely related *C. difficile* strains. It is also of interest that these lineages of Clade 2 appear to be associated with the largest degree of TcdB subtype diversification, with four different TcdB subtypes (TcdB2, 4, 7, and 9), and they show a phylogenetically incongruent distribution on the tree, consistent with recombination events (Supplementary Fig. 14a).

Our previous phylogenetic analysis of all *tcdB* genes has suggested a putative evolutionary path for the origin of TcdB subtypes^50^: a TcdB ancestor split into two precursor toxins: a TcdB1-like (type i), which diversified into TcdB1/5/6, and a TcdB7-like toxin (type ii), which diversified into TcdB4/7 (Supplementary Fig. 14b). TcdB2/9/3/8 were generated through recombination events between the TcdB7-like precursor toxin and members of the TcdB1-like lineage. Based on this evolutionary path, TcdB2.1 is a recombinant subtype that was generated by fusion of an *ancestral* type i N-terminal fragment, with an *ancestral* type ii C-terminal fragment including the DRBD and CROPs domain. Our discovery of the unique B4/B7 haplotype, which is conserved in TcdB4 and members of TcdB7, but missing from TcdB2, is consistent with a model in which TcdB2 split from TcdB4/7 prior to their acquisition of this TFPI-binding haplotype. Moreover, the residues in TcdB2.1 corresponding to B4/B7-haplotype residues are all identical to the residues found in more distantly related TcdB1.1, further suggesting that the TFPI-binding B4/B7-haplotype is an evolutionarily recent gain-of-function event. It remains possible that TcdB2.1 may have low-affinity interactions with TFPI on cell surfaces. In this case, TcdB2.1 and its ancestor toxins could be an evolutionary “steppingstone” towards evolving high-affinity interactions with TFPI. It is also possible that TcdB2.1 may recognize specific TFPI orthologs from certain host species that we did not test, or even an unidentified receptor unrelated to TFPI or FZDs.

Because the key TFPI-binding substitutions occur as a linked set of substitutions (a haplotype), it is challenging to tease apart the precise order in which these substitutions occurred and the details of the evolutionary transition toward TFPI-binding. Nevertheless, our data suggest that certain substitutions may serve as key “driver” substitutions, which are potentially responsible for the initial shift toward TFPI specificity in TcdB4/7. One such substitution is that of S1495, which is at the center of a β-strand in the FZD/TFPI/SEMA6 interface. This residue, S1495 on TcdB1 and R1495 on TcsL, directly interacts with FZDs and SEMA6, respectively^27,39^.

We showed that TFPI is a relevant receptor mediating functional entry of TcdB4 into human intestinal organoids (including undifferentiated enteroids and differentiated rectoids) and mouse intestinal organoids. We note that these organoids are in 3D culture, and we have not tested the role of TFPI in monolayer culture of human organoid cells, which is a limitation of our study. TFPI is well known to be expressed on blood vessel endothelial cells, as well as monocytes, smooth muscle cells, and platelets^64^. Interestingly, the receptors for TcsL, SEMA6A and 6B, are also expressed in endothelial cells. Systemic administration of TcsL causes damage mainly to lung tissues, which is mediated by SEMA6A and 6B^38,39,71^. Consistent with this, we observed that systemic administration of TcdB4 also led to severe damage to lung tissue in vivo in mouse models, which was mediated by TFPI since excessive recombinant TFPI fragments reduced the toxicity of TcdB4 on lung tissue in vivo.

TcdB10/11/12 appears to be early diverging lineages from an ancestral TcdB toxin^50^. This is consistent with the finding that the TFPI-binding B4/B7-haplotype is not found in TcdB10/11/12. TcdB10 and TcdB4/7 appear to have evolved TFPI binding capability independently, illustrating the remarkable ability of this receptor-binding region for developing protein-protein interactions. The selective pressure toward TFPI as a receptor remains to be established. TcdB10, 11, and 12 all were recently identified from highly divergent cryptic genomospecies, which have been proposed to predate *C. difficile* by millions of years^72^. They are associated with rare clinical cases (two for TcdB10, two for TcdB11, and one for TcdB12) and some of these toxins are encoded on mobile genetic elements^54–56^. Their prevalence might have been underestimated, as Clade C-I strains represent a diagnostic challenge due to the high sequence and likely antigenic divergence of their toxins. Whether these rare subtypes may represent toxins that normally affect certain animal species with only occasional transmission to humans remains to be further explored.

In summary, our studies establish the receptor binding specificity for TcdB subtypes from clinical strains and identify TFPI as a receptor replacing FZD1/2/7 for several TcdB variants. The origin of TFPI specificity can be traced to specific amino acid substitutions as a haplotype within the previously determined FZD-binding interface, which can be considered more generally as a variable receptor-specificity determining region. Our identification of receptor-switching in TcdB clearly demonstrates that IR between different lineages, during either co-infection or co-existence in their natural reservoir, can be a key mechanism for rapid divergence and exchange of functional specificity for pathogens. IR has the potential to rapidly alter gene function, especially when a gene is organized in a modular way in which different segments may carry their own distinct functions that can be transferred to recipient genes through recombination. Therefore, IR at a highly adaptable specificity-determining region can be an evolutionarily favored mechanism to rapidly generate protein sequence variations and explore functional diversity without the risk of disrupting the overall structure and fold^73^, which could be critical for emerging and re-emerging pathogens to rapidly sample multiple adaptation routes and a range of tissue/host tropism over a short evolutionary time-scale.

**Note:** A related work by Luo et al.^74^ was published during the review of this manuscript. Luo et al. identified TFPI as a receptor for TcdB4.1 using CRISPR-Cas9 genome-wide screening in HeLa cells and solved a cryo-EM structure of the TcdB4-TFPI complex. The structure reveals a binding interface that is consistent with our mutagenesis analysis reported here. Luo et al. reported that TcdB2 can utilize TFPI as a receptor, whereas our data suggest that only one member of TcdB2 (TcdB2.11) can recognize TFPI, and other TcdB2 members do not utilize TFPI as a receptor. In addition, Luo et al. reported a pathogenic effect of TcdB4.1 in a mouse colon loop ligation model, whereas we showed a lack of pathogenic effect of TcdB4.2 in cecum injection assays in mice.

## Methods

### Cell lines, bacterial strains, mice, and antibodies

The following cell lines were all originally obtained from ATCC with their catalog number noted: HeLa (CCL-2), A549 (CRM-CCL-185), HEK293T (CRL-3216), and 5637 (HTB-9). The HeLa-Cas9 cell line was generously provided by Dr. Abraham Brass (Worcester, MA). All cells were cultured in DMEM media plus 10% fetal bovine serum (FBS) and 100 U penicillin / 0.1 mg/mL streptomycin in a humidified atmosphere of 95% air and 5% CO_2_ at 37 °C. Expi293F was purchased from Thermo Fisher (A14527) and cultured in FreeStyle 293 Expression Medium (12338026). HUVECs were purchased from Lonza (00191027) and cultured in F-12K media contains 10% FBS, 0.1 mg/mL heparin, and endothelia cell growth supplement (ECGS). *C. difficile* strains were cultured in Brain Heart Infusion Broth (ThermoFisher, CM1135B) in anaerobic chamber, and their source information is listed in Supplementary Table 1. All animal studies, including euthanasia via carbon dioxide asphyxiation, were conducted according to ethical regulations under protocols approved by the Institute Animal Care and Use Committee (IACUC) at Boston Children’s Hospital (18-10-3794 R). Housing conditions: 12 h dark/light cycle, 25 °C, 30–40% humidity. CD1 strain mice were purchased from Charles River. Antibodies were obtained from the indicated vendors: Actin (Aves Labs, ACT-1010), mouse anti-HA tag (BioLegend, 901502), chicken anti-HA tag (Aves Labs, ET-HA100), mouse anti-FLAG tag (Sigma, F3165), rabbit anti-FLAG tag (Abcam, ab205606), 1D4 tag (ThermoFisher, MA1-722), TFPI (Abcam, ab260042), and TcdB (List Bio, 754 A).

### cDNA constructs

The selected sgRNA sequences (UGP2: ATCCTGCATTAAGACTATAG; TFPI: ATATAACCTCGACATATTCC; TFPI2: TGTGATGCTTTCACCTATAC; PIGS: GATCTGGGAGTAAGGCAACG; PIGV: TGGTGAAAGGATGTGGCCCC) were cloned into the LentiGuide-Puro vector (Addgene, #52963). The cDNA encoding the regions 1285-1804 of TcdB4.2, 2.1, 2.11, 7.2, 7.5, and 11.2, were synthesized, and codon-optimized for *E. coli* expression (Twist Bioscience). Full-length TcdB2.2, 4.2, 7.1, 7.2, 12.1, TcsL, and TcdB2.1_1–__1833_ were subcloned into the pHis1522 vector with a C-terminal 6xHis tag (MoBiTec GmbH). TcdB4.2_1286–__1805_, TcdB4.2_1835–__2367_, TcdB2.1_1285–__1804_, TcdB2.11_1286–__1805_, TcdB7.2_1286–__1805_, TcdB7.5_1286–__1805_, TcdB11.2_1285–__1804_, TcdB4.2(B1.1), and TcsL_1285–__1804_ were cloned into the pET28a vector (Novagen) with a 3xFLAG tag and a 6xHis tag at their C-termini via Gibson Assembly (NEB, E2621). TcdB1.1(B4.2) and TcdB1.1-FBD-5M were cloned into the pET28a vector with a HA tag at its N-termini and a 6xHis tag at its C-termini. The genomic DNA of *C. difficile* strains CD10-165 and 173070 was extracted. The DNA fragments of TcdB10.1_1285–__1804_ and TcdB12.1_1285–__1804_ were amplified by PCR and cloned into the pET28a vector fused to the diphtheria toxin enzymatic domain and translocation domain (DTAT, residues 3–378) at their N-termini and a 6xHis tag at their C-termini. TcdB2.1_1285–__1804_-MT1, TcdB2.1_1285–__1804_-MT23, TcdB2.1_1285–__1804_-MT4, TcdB2.1_1285–__1804_-MT56, TcdB2.1_1285–__1804_-MT123, TcdB2.1_1285–__1804_-MT1234, TcdB2.1_1285–__1804_-MT12356, TcdB2.1_1285–__1804_-MT23456, TcdB2.1_1285–__1804_-MT123456, TcdB11.2_1285–__1804_-MT12, TcdB11.2_1285–__1804_-MT34, and TcdB11.2_1285–__1804_-MT1234 were generated by site-directed mutagenesis via QuikChange kit (Agilent, #200518). The cDNAs of TFPI were obtained from the indicated vendors: TFPI (Horizon Discovery, MHS6278-202756867), TFPI2 (Horizon Discovery, MHS6278-202839472), and mouse TFPI (Sino Bio, MG50131-M). The cDNA of cattle TFPI, chicken TFPI, and dog TFPI were synthesized and codon-optimized to human expression (Genewiz). TFPI (residues 29-209), TFPI2 (residues 23–235), and mouse TFPI (residues 29–217) were cloned into the pLenti-Hygro vector (Addgene, #17484) with the PDL1 signal and a 3xHA tag (with EFGSGSGS linker) at their N-termini and TFPI β domain (residues 210–251) at their C-termini. TFPI (residues 29–209, 29–124, or 105–209), TFPI2 (residues 23–235), mouse TFPI (residues 29–217), cattle TFPI (residues 25–208), chicken TFPI (residues 29–212), and dog TFPI (residues 29–209) were cloned into pcDNA3.1 vector (Invitrogen, V800-20) with the IL-2 signal at their N-termini (with GGGGGGR linker) and a human Fc-Myc-6xHis tag (with EFGSGSGS linker) at their C-termini. 1D4-tagged full-length mouse FZD2 was obtained from Addgene (#42264).

### Recombinant proteins

Recombinant His-tagged TcdB1.1, 2.1, 2.2, 4.2, 7.1, 7.2, 12.1, TcdB2.1_1–__1833_, and TcsL were expressed in *B. megaterium* following the supplier’s protocol (MoBiTec GmbH). The fragments of TcdB variants and their mutations were expressed in *E. coli* (BL21 strain) and purified as His-tagged proteins. Recombinant human Fc-tagged chimera proteins were purchased from R&D Systems: SEMA6A-Fc (1146-S6), CRD2-Fc (1307-FZ), and IgG-Fc (110-HG). Recombinant TFPI-Fc, TFPI2-Fc, mTFPI-Fc, Cattle TFPI-Fc, Chicken TFPI-Fc, Dog TFPI-Fc, TFPI-K1-Fc, and TFPI-K2-Fc proteins were expressed using Expi293F cells (Life Technologies). Briefly, 3 × 10^7^ Expi293F cells were transfected with 37.5 µg plasmid using PEIMax (1 mg/mL) (Polysicences). The culture medium was harvested 5 days after transfection. The proteins in the culture medium were collected and purified as His-tagged proteins.

### Native *C. difficile* culture supernatants

*C. difficile* strains were streaked on Brain Heart Infusion agar plates (BD, 297848) and incubated in an anaerobic chamber at 37 °C for 24 h. Single clones for each strain were inoculated in Brain Heart Infusion Broth (ThermoFisher, CM1135B) and cultured in an anaerobic chamber for 36–48 h. Cultures were then 1:100 inoculated to Cooked Meat Medium (VWR, 90001-914) for most strains and TYT broth for strains expressing TcdB7.9 and TcdB11.2. They were incubated in an anaerobic chamber at 37 °C for 3–5 days. After centrifugation at 5000 × *g* for 10 min, the culture supernatants were collected, filtered through a 0.22 μm filter, and stored at −80 °C.

### Cell-rounding assay

The cytopathic (cell-rounding) effect of TcdB subtypes was analyzed using a standard cell-rounding assay. Briefly, cells were seeded into 96-well plates and exposed to toxins or native culture supernatants at the indicated concentration or dilution and time. A chicken polyclonal antibody (List Bio, 754 A) was used to neutralize TcdB. The phase-contrast images were taken (Olympus IX51, 10–20 × objectives). A zone containing 50–200 cells (~300 × 300 µm) was selected randomly, and round-shaped and normal-shaped cells were counted manually. The percentage of round-shaped cells was analyzed using the OriginPro (OriginLab, v8.5) and Excel (Microsoft, 2007) software. Data were represented as mean ± s.d. from three independent biological replicates. Data were considered significant when *p*-value <0.05 (Student’s *t*-test).

### Genome-wide CRISPR-Cas9-mediated genetic screens for TcdB4

CRISPR-Cas9 mediated genetic screens were performed as previously described^38,75^. Briefly, the GeCKO-V2 sgRNA library was obtained from Addgene (#1000000049)^76^. The sub-library A and B were independently packed into lentiviral libraries. HeLa-Cas9 cells were transduced with sgRNA lentiviral library at a MOI (multiplicity of infection) of 0.2. Polybrene (Santa Cruz, sc-134220, 8 μg/mL) was added to the medium to facilitate viral transduction. The infected cells were selected with Puromycin (Thermo Scientific, A1113830, 5 µg/mL). To ensure sufficient sgRNA coverage, 3.3 × 10^7^ and 2.9 × 10^7^ cells were plated in 15-cm culture dishes for sub-library A and B, respectively (500 × coverage, each sgRNA being represented 500 times). These cells were exposed to the culture supernatant of TcdB4.2 for 3 d (with fresh toxin-containing medium replaced daily). The surviving cells were washed and re-seeded within toxin-free medium until ~70% confluence, followed by the next round of selection. In total three rounds of selections were performed with 1/500,000, 1/250,000, and 1/125,000 dilution (v/v) of TcdB4 supernatant, respectively. The genomic DNA of untreated cells (Ctrl gDNA) and final surviving cells (TcdB4.2 gDNA) was extracted using a commercial kit (Qiagen, 13323). DNA fragments containing the sgRNA sequences were amplified by PCR using primers lentiGP-1_F (AATGGACTATCATATGCTTACCGTAACTTGAAAGTATTTCG) and lentiGP-3_R (ATGAATACTGCCATTTGTCTCAAGATCTAGTTACGC). Next-generation sequencing was performed by a commercial vendor (Genewiz, Illumina MiSeq).

### Generating KO and overexpression cells via lentiviral transduction

A549 cells that stably express Cas9 were generated using LentiCas9-Blast (Addgene, #52962) and selected using 10 µg/mL Blasticidin S (RPI, B12150.01). HeLa-Cas9 and A549-Cas9 cells were utilized for generating KO cells via lentiviral transduction of sgRNAs. Mixed populations of infected cells were selected with Puromycin (10 µg/mL for A549, and 5 µg/mL for HeLa, respectively). Notably, cell population remain as a mixture with multiple genotypes and variable knockout efficacies. Single clones of CSPG4-KO cells were generated by diluting the mixed KO cells at around 0.8 cell per well in 48-well plates. The single clones were selected and their genotypes were determined by amplifying the DNA fragments containing the sgRNA targeting region by PCR using primers CSPG4-GT_F (CGATGCCTTCTCGCTGGATGT) and CSPG4-GT_R (GTGCTTCTGAAATGTGACTCCCCGT), followed by ligating the PCR product into T-vectors (Promega, A3600). The ligation products were transformed into *E. coli* (DH5a strain) and plated onto agar plates. Twenty *E. coli* colonies were selected, and their plasmids were extracted and sequenced. HeLa and 5637 cells were utilized by transduction with lentiviruses expressing TFPI proteins, and cells were selected with 200 µg/mL Hygromycin B (EMD Millipore, 400051).

### Immunoblot analysis

Cells were scraped, washed, and lysed with RIPA buffer (50 mM Tris, pH 7.5, 1% NP-40, 150 mM NaCl, 0.5% sodium deoxycholate, 1% SDS, protease inhibitor cocktail) on ice for 30 min. Protein amounts in cell lysate were measured by a BCA assay (Thermo Scientific, 23225). Cell lysates were mixed with SDS-PAGE loading buffer (50 mM Tris, pH 6.8, 2% SDS, 10% glycerol, 0.01% bromophenol blue, 20 mM DTT), heated for 5 min, analyzed by SDS-PAGE, and transferred onto a nitrocellulose membrane (GE Healthcare, 10600002). Membranes were blocked with TBST buffer (10 mM Tris, pH 7.4, 150 mM NaCl, 0.1 % Tween-20) containing 5% skim milk at room temperature for 40 min. The membrane was then incubated with the primary antibodies (1:1000 dilution) for 1 h, washed, and incubated with secondary antibodies (1:2000 dilution) for 1 h. Signals were detected using the enhanced chemiluminescence method (Thermo Fisher Scientific, 34080) with a Fuji LAS3000 imaging system. Images were analyzed and quantified using ImageJ software (Version 1.52o)

### Biolayer interferometry (BLI) assay

The binding affinities (*K*_D_) of TcdB variants and TFPI proteins were measured using a BLI assay with the BLItz system (ForteBio) and calculated using the BLItz system software. Briefly, 10 μg/mL Fc-tagged proteins were immobilized onto capture biosensors (Dip and Read Anti-Human IgG Fc Capture, ForteBio) and balanced with DPBS (0.5% BSA, w/v). The biosensors were then exposed to 500 nM or the indicated concentrations of full-length TcdB variants, toxin fragments, or their mutations, followed by dissociation in DPBS (0.5% BSA, w/v).

### Toxin cell surface binding and immunostaining

HeLa cells were transfected with the indicated constructs by PolyJet reagent (SignaGen, SL100688), seeded onto glass coverslips (Hampton, HR3-239) in 24-well plates, and incubated for 48 h until ~70% confluence. Cells were washed three times with ice-cold PBS, and incubated with 5 µg/mL TcdB4.2_1286–__1805_-FLAG in medium on ice for 60 min. Cells were washed, fixed with 4% paraformaldehyde (PFA) for 20 min at room temperature, permeabilized with 0.3% Triton X-100 for 30 min, and blocked with 10% goat serum for 40 min, followed by incubation with primary antibodies (1:1000 dilutions) for 1 h and fluorescence-labeled secondary antibodies (anti-Rabbit Alexa-488, ThermoFisher, # A-11008, anti-Mouse Alexa-546, ThermoFisher, # A-11030, 1:2000 dilutions) for 1 h. Slides were sealed within DAPI-containing mounting medium (SouthernBiotech, 0100-20). Fluorescent images were captured using an Olympus DSU-IX81 Spinning Disk Confocal System. Images were pseudo-colored and analyzed using ImageJ.

### In vitro competition assays

Toxins (4 pM TcdB4.2, 4 pM TcdB7.1, 4 pM TcdB7.2, or 40 pM TcdB12.1) or culture supernatants (1/10 dilution of TcdB7.9, 1/100 dilution of TcdB10.1, or 1/1000 dilution of TcdB11.2) were pre-mixed with or without recombinant Fc-tagged TFPI, TFPI2 or mTFPI at indicated ratio/concentration in culture medium and incubated on ice for 1 h. The mixtures were then added to cells. Cells were further incubated at 37 °C and the percentages of cell rounding at indicated time points were examined.

### Factor Xa activity assay

Competition of TcdB4.2 against TFPI for its natural ligand coagulation factor Xa (FXa) was performed using a commercial kit (Sigma, MAK238-1KT). In brief, FXa (0.5 ng/μL), TFPI (5 ng/μL TFPI-Fc, 5 ng/μL mTFPI-Fc, or 7.5 ng/μL TFPI-K2-Fc), and TcdB fragments (at indicated concentrations) were mixed with FXa’s fluorescently labeled substrate in the assay buffer, as indicated following the vendor’s protocol. Substrate cleavage generates increasing fluorescent signal with time, which was measured as relative light unit (RLU) by a microplate reader (BioTek, Synergy Neo2). FXa activity was quantified by measuring the slope of RLU curves (1–10 min) using Excel software (Microsoft).

### Human and mouse intestinal organoids

Mouse intestinal organoids were derived from duodenum of C57BL/6 mice. Briefly, about 10 cm of proximal duodenum was harvested, opened longitudinally, and washed with cold PBS to remove luminal content. The tissue was then cut into 5 mm pieces with a new sterile razor blade and further washed 5–10 times with cold PBS. Tissue fragments were incubated with 2 mM EDTA in PBS for 15 min on ice. After removal of EDTA, tissue fragments were replaced with fresh 2 mM EDTA and incubated for another 25 min on ice. These fragments were then shaken vigorously for 1 min and further triturated with a 10 mL serological pipet to release crypts. Supernatant fractions enriched in crypts were collected, passed through a 70 μm cell strainer, and centrifuged at 300 × *g* for 5 min. The cell pellet was then washed three times in DMEM/F12, centrifuged at 300 × *g* for 5 min. Crypts were then resuspended in 200–300 μL of Matrigel (Corning, 356231) with 50 μL per well on 24-well plates and polymerized at 37 °C. The crypts were grown in Matrigel with mouse organoid growth medium, which contains (v/v): Rspondin-1 conditioned media (10%), DMEM/F12 (85%), Glutamax (1%), N-2 supplement (1%), B-27 supplement (1%), HEPES (10 mM), primocin (100 µg/mL), normocin (100 μg/mL), N-acetyl-cysteine (1.25 mM), recombinant murine Noggin (100 ng/mL), and recombinant murine EGF (50 ng/mL).

Cultured human duodenal and rectal organoids, enteroids and rectoids, respectively, were provided as de-identified materials from the Harvard Digestive Disease Center organoid core facility. Human organoids were originally from de-identified biopsy samples from pediatric patients undergoing esophagogastroduodenoscopy and colonscopy at Boston Children’s Hospital. All methods were approved by the Institutional Review Board of Boston Children’s Hospital (Protocol number IRB-P00000529). To isolate crypts, biopsies were digested in 2 mg/mL of Collagenase Type I (Life Technologies, 17018029) reconstituted in Hank’s Balanced Salt Solution for 40 min at 37 °C. Samples were then agitated by pipetting followed by centrifugation at 500 *g* for 5 min at 4 °C. Isolated crypts were grown in Matrigel with organoid growth medium based on the tissue of origin. The growth medium for duodenal organoids contains (v/v): L-WRN conditioned media (50%), DMEM/F12 (45%), Glutamax (1%), N-2 supplement (1%), B-27 supplement (1%), HEPES (10 mM), primocin (100 µg/mL), normocin (100 μg/mL), A83-01 (500 nM), N-acetyl-cysteine (500 μM), recombinant murine EGF (50 ng/mL), human [Leu15]gastrin I (10 nM), nicotinamide (10 mM), and SB202190 (10 μM). The growth medium for rectal organoids contains (v/v): L-WRN conditioned media (65%), DMEM/F12 (30%), Glutamax (1%), N-2 supplement (1%), B-27 supplement (1%), HEPES (10 mM), primocin (100 µg/mL), normocin (100 μg/mL), A83-01 (500 nM), N-acetyl-cysteine (500 μM), recombinant murine EGF (50 ng/mL), human [Leu15]gastrin I (10 nM), nicotinamide (10 mM), SB202190 (10 μM), and Prostaglandin-E2 (10 nM).

Human and mouse growth media were changed every 2–3 days. After 6–8 days of culture, media was removed, and Cell Recovery Solution (Corning, 354253) was added. The plate was incubated at 4 °C for 1 h. The Matrigel was mechanically resuspended and centrifuged at 500 × *g* at 4 °C for 5 min. The pelleted organoids were resuspended in fresh Matrigel and mechanically disrupted by pipetting up and down. The suspension was seeded into a fresh 48-well plate at 25 μL per well. After incubation at 37 °C for 10 min, 250 μL of pre-warmed organoid growth medium was added.

Human rectal organoids were grown and differentiated as previously described^68^. Briefly, rectal organoids were passaged as above, and grown in growth medium for two days, after which the rectoids were transitioned to differentiation medium which contains (v/v): L-WRN conditioned media (65%), DMEM/F12 (30%), Glutamax (1%), N-2 supplement (1%), B-27 supplement (1%), HEPES (10 mM), primocin (100 µg/mL), normocin (100 μg/mL), A83-01 (500 nM), N-acetyl-cysteine (500 μM), recombinant murine EGF (50 ng/mL), human [Leu15]gastrin I (10 nM), DAPT (20 μM), Betacellulin (20 ng/mL), Tubastatin A (10 μM), PF06260933 (6 μM), and Tranylcypromine (1.5 μM). Media was changed every two days, with Tubastatin A being removed after the second day of differentiation.

After three days in culture for mouse organoids and human enteroids, or 14 days for differentiated rectoids, TcdB4.2 alone (10 pM) or TcdB4.2 pre-incubated with Fc-tagged TFPI, TFPI2, or mTFPI at 100 nM (1:10,000 molar ratio) were added to the organoids for 8 h treatment. We defined “intact organoids” as organoids with a normal morphology (spheroid-shape for undifferentiated human organoids and the presence of crypt-like buds for differentiated human organoids and mouse organoids) and an intact epithelial layer. The intact organoids were counted, and their size (longest diameter) was measured under a microscope. Then, organoids were cultured for three more days. MTT (0.5 mg/mL, Research Products International, M92050) was added to each well and incubated for 4 h at 37 °C. The medium was removed and 200 μL solubilization solution (10% SDS in 0.01 M HCl) was added to each well, incubated overnight at room temperature, and the absorbance of formazan was measured at 580 nm using a microplate reader (BMG Labtech, FLUOstar Omega). A vehicle control without toxin treatment was analyzed in parallel.

### Cecum-injection assay

Adult CD1 mice (8–10 weeks of age, 17–20 g bodyweight, female) were anesthetized with 3% isoflurane after overnight fasting. A midline laparotomy was performed. Saline solution, TcdB1.1 or TcdB4.2 at indicated doses was injected into the cecum through the ileocecal junction. The gut was then returned to the abdomen. The incision was closed with stitches and mice were allowed to recover. After 6 h, mice were euthanized, and the cecum tissue was harvested. Cecal luminal contents were extracted using 1 mL PBS, filtered through a 0.45 μm filter, and used to perform cell rounding assays. The cecum was fixed with 10% phosphate buffer formalin and embedded in paraffin. Tissue sections were subjected to hematoxylin and eosin (H&E) staining for histological analysis.

### Limited proteolysis assay

The limited trypsin digestion assays were performed on TcdB1.1 and TcdB4.2 in pH 7.8 buffer (20 mM HEPES, 250 mM NaCl) and pH 5.8 buffer (20 mM sodium citrate, 250 mM NaCl). Toxins (0.5 mg/mL) were mixed with trypsin at 50:1 molar ratio, and the reactions were incubated at room temperature (20 °C). Samples taken at the indicated time were mixed with SDS-PAGE loading buffer (with 20 mM DTT) and boiled for 5 min to quench the reaction, then examined by SDS-PAGE and visualized using Coomassie blue staining.

### In vivo toxicity assays

Adult CD1 mice (8–10 weeks of age, 17–20 g bodyweight, male and female, randomly separated into experimental groups) were injected with TcdB4.2 or TcdB 1.1 (50 ng/25 g bodyweight, diluted in 100 µL saline) pre-incubated with or without Fc-tagged TFPI, TFPI2, or mTFPI (1/2000 molar ratio) via IP. Saline was used as a control. Mice were euthanized 4 h or 15 h after the injection to first collect the fluid in the thoracic cavity, then lung tissues were harvested and weighed (wet weight). The tissues were then dried in an oven at 100 °C overnight and weighted (dry weight). The ratios of dry-to-wet weights of lung tissues were calculated for each mouse. Portions of freshly harvested lung tissues were fixed with 10% formalin in phosphate buffer and embedded in paraffin. Tissues were sectioned and histological analysis was carried out after H&E staining.

### Knockdown TFPI by RNAi

The TFPI targeting siRNA was purchased from Santa Cruz (sc-41060). The non-targeting scramble siRNA was purchased from Life Technologies. HUVECs were seeded in 96-well plates for 24 h. When confluency reached 70%, cells were incubated in serum-free medium for 8 h. The siRNAs were transfected into cells using Lipofectamine RNAiMAX (ThermoFisher). Experiments were carried out 48 h later. The knockdown efficiency was validated by immunoblot analysis.

### Bioinformatic analyses

Sequence analysis: All available unique sequences of TcdB and TcsL (*N* = 212) were downloaded from the *DiffBase* database^50^. These sequences were aligned as described previously (Mansfield et al., 2020), and imported into R v4.1.1 using the seqinr v4.2-8 and BALCONY v0.2.10 packages^77^ to generate an alignment data matrix. Amino acid variation at specific positions of interest (1495,1505,1506,1511,1547,1596,1597,1598,1599,1602; numbering based on TcdB1.1) was then visualized using ComplexHeatmap v2.8.0^78^.

Haplotype visualization: Haplotype visualization was performed as described previously (Mansfield et al., 2020) using the haploColor algorithm available at https://github.com/doxeylab/haploColor. Briefly, the sequence alignment was first converted to a 2D data matrix. All sequences were then colored black at all positions matching the first reference sequence (TcdB1.1), remaining (non-colored) positions were colored green where they matched a second sequence (TcdB2.1), and then remaining (non-colored) positions were colored red where they matched a third sequence (TcdB4.2). Finally, remaining uncolored residues were assigned a gray color.

Recombination detection: To visualize intragenic recombination events, we performed a sliding window analysis of pairwise sequence identities. Query sequences of interest (B2.11, B7.11, and B7.2) were aligned to target sequences (B1.1, B2.1, B4.2, and B7), and percentage identities were calculated for sliding windows of window length = 50 amino acids. Pairwise identities for each query-target pair were plotted and colored uniquely across the full-length of the toxin, which facilitated visualization of recombinant regions.

### Structural analysis

Structural visualization and modeling were performed using PyMol v2.4.1. The TcdB1-FZD interface was analyzed using PDB ID 6C0B, and the B4/B7 haplotype residues and B10-specific substitutions were made using PyMol’s mutagenesis function and the default rotamer. The TcsL-SEMA6 interface was analyzed using PDB ID 6WTS.

### Statistical analysis

Data were considered statistically significant when *p* < 0.05 using Student’s *t*-test (two-sided) as indicated in the Figure legends. Data were represented as mean ± s.d. from at least three independent biological replicates. Statistical analysis was performed using OriginPro and Excel software.

### Reporting summary

Further information on research design is available in the Nature Research Reporting Summary linked to this article.

## Supplementary information

Supplementary Information

Description of Additional Supplementary Files

Supplementary Data 1

Reporting Summary

## Source data

Source Data

## Data Availability

DiffBase database (https://diffbase.uwaterloo.ca/) for downloading TcdB sequences. HaploColor algorithm (https://github.com/doxeylab/haploColor) for haplotype visualization. Expression of TFPI in various lung tissue cells were plotted based on published single cell RNAseq data (http://betsholtzlab.org/VascularSingleCells/database.html). The list of CRISPR-Cas9 screening results generated in this study are provided in the Supplementary Data 1. The raw data for each figure in both main text and the Supplementary Information is presented in the Source Data (Excel file, one figure per sheet). All data and materials used in the study are available to any researcher for purposes of reproducing or extending the analysis. Source data are provided with this paper.
