# Supplementary material for: Identification of TFPI as a receptor reveals recombination-driven receptor switching in Clostridioides difficile toxin B variants
Source: Nat Commun. 2022 Nov 9;13:6786. doi: 10.1038/s41467-022-33964-9 (PMC9646764; doi:10.1038/s41467-022-33964-9)
Supplement: Supplementary file 1 — Supplementary Information [file 41467_2022_33964_MOESM1_ESM.pdf]

Supplementary Information

Supplementary Figures 1-14

Full scans for immunoblots

Supplementary Table 1: Information on *C. difficile* strains used in this study.

Supplementary Data1: Excel file listing CRISPR-Cas9 screening results.

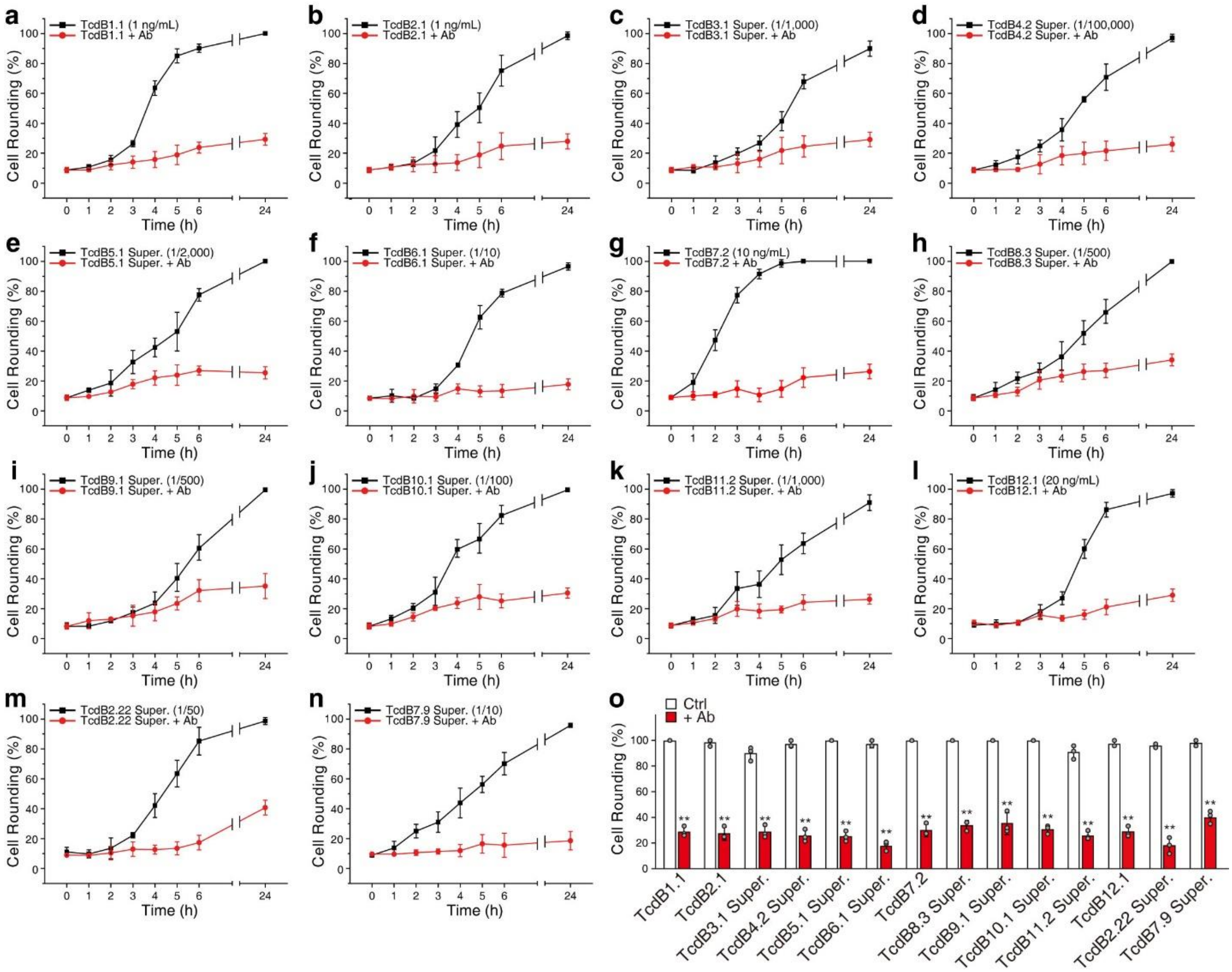

Supplementary Figure 1: Cell-rounding activity in *C. difficile* culture supernatants can be neutralized with a polyclonal anti-TcdB antibody.

a-n HeLa-WT cells were exposed to either recombinantly purified TcdB1.1 (**a**), TcdB2.1 (**b**), TcdB7.2 (**g**), and TcdB12.1 (**l**), or culture supernatants from *C. difficile* strains expressing TcdB3.1 (**c**), TcdB4.2 (**d**), TcdB5.1 (**e**), TcdB6.1 (**f**), TcdB8.3 (**h**), TcdB9.1 (**i**), TcdB10.1 (**j**), TcdB11.2 (**k**), TcdB2.22 (**m**), and TcdB7.9 (**n**), with or without a polyclonal anti-TcdB antibody (+ Ab). Cell rounding was quantified over time. Error bars indicate mean ± s.d.; $N = 3$ (biologically independent experiments).

o Experiments were carried out as described in panels **a-n**, the degrees of cell-rounding with 24 h incubation were plotted as a bar-chart. Error bars indicate mean ± s.d.; $N = 3$ (biologically independent experiments); **, $p < 0.01$ (Student's *t*-test, two-sided).

Source data are provided as a Source Data file.

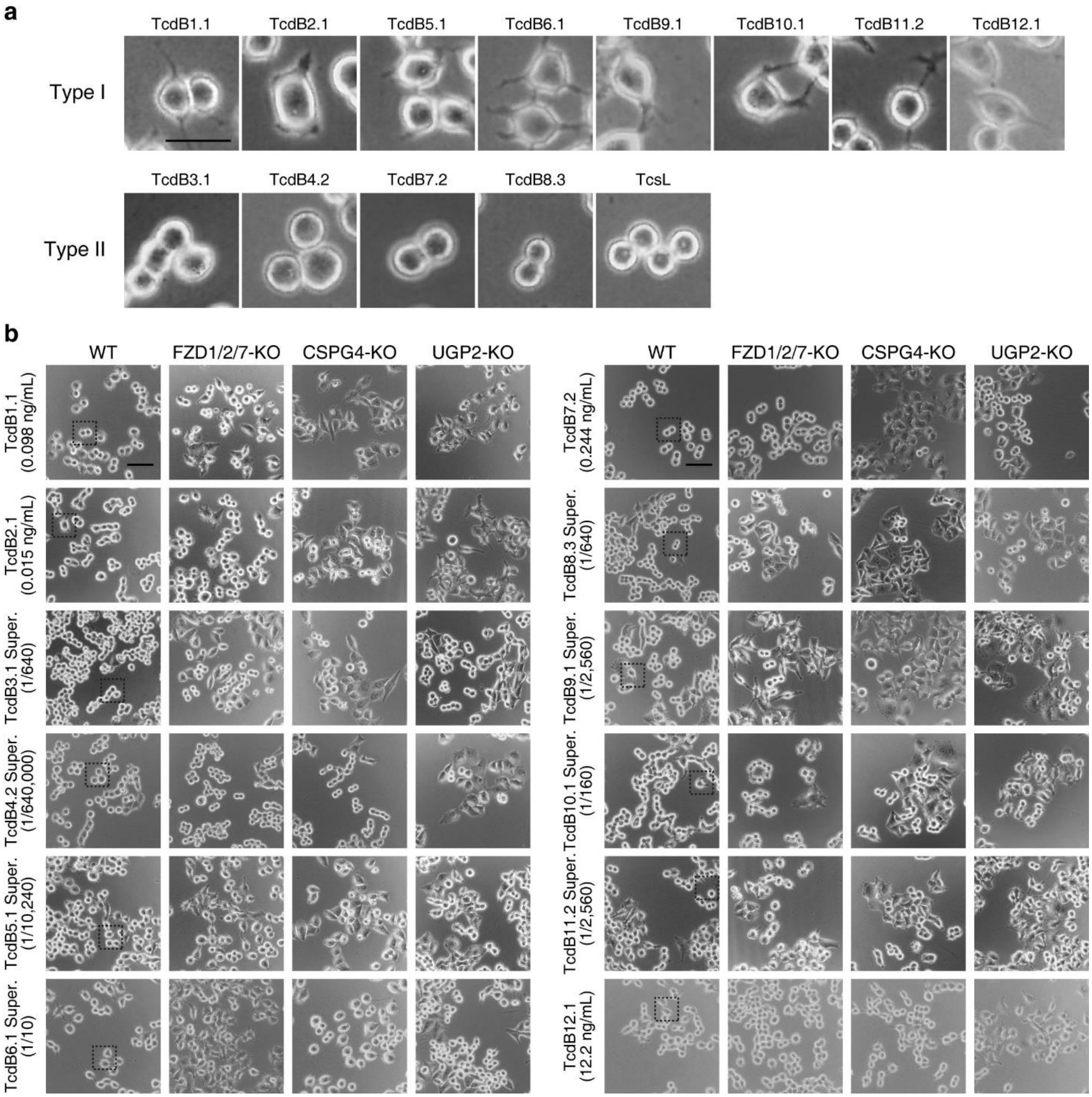

Supplementary Figure 2: Representative images showing the cytopathic phenotype induced by TcdB subtypes.

a Representative images showing that TcdB variants induce two types of morphological changes in HeLa-WT cells. Type I includes TcdB1.1, TcdB2.1, TcdB5.1, TcdB9.1, TcdB10.1, TcdB11.2, and TcdB12.1. Type II includes TcdB3.1, TcdB4.2, TcdB7.1, and TcdB8.3. TcsL also induces type II morphotype. Scale bar, 10 µm.

b Representative images showing the cell rounding effect in HeLa-WT, FZD1/2/7-KO, CSPG4-KO, and UGP2-KO cells after incubation with the indicated concentrations of TcdB variants for 24 h. Scale bar, 20 µm. Dash boxes in WT cells indicate the representative cells shown in panel **a**. Representative images were from one of three independent experiments.

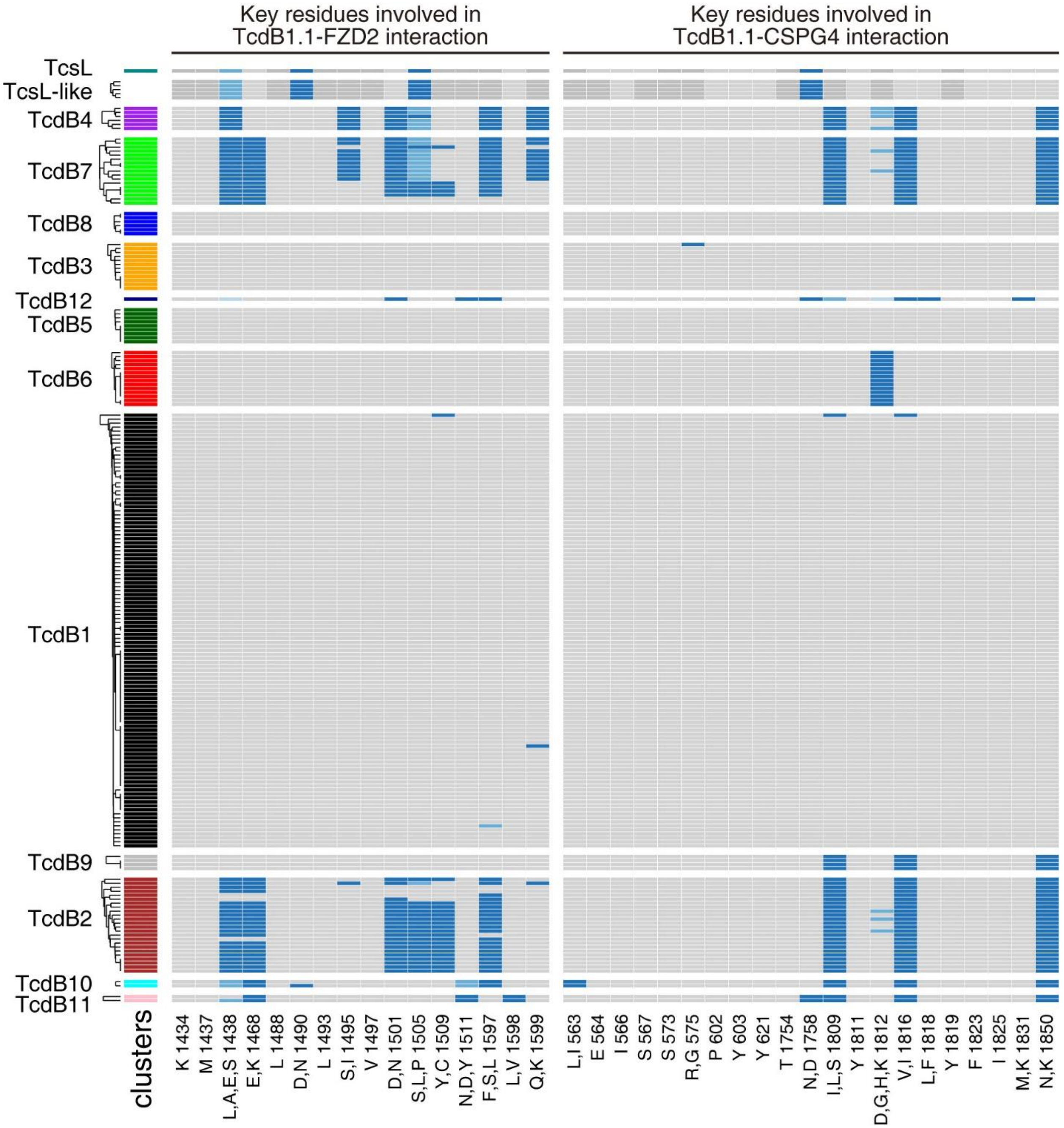

Supplementary Figure 3: Key residues involved in TcdB1.1-FZD2 and TcdB1.1-CSPG4 interactions across all known TcdB sequences.

Alignment of key positions for FZD interactions and CSPG4 interactions across all 206 TcdB sequences and 6 TcsL family sequences. Residues that are shared in TcdB1.1 are colored gray. Variable residues are colored blue (darkest blue = most common variant). Sequence ordering is based on phylogenetic analysis of full-length protein sequences. FZD-binding residues are based on PDB 6C0B; and the CSPG4-binding residues are based on PDB 7ML7.

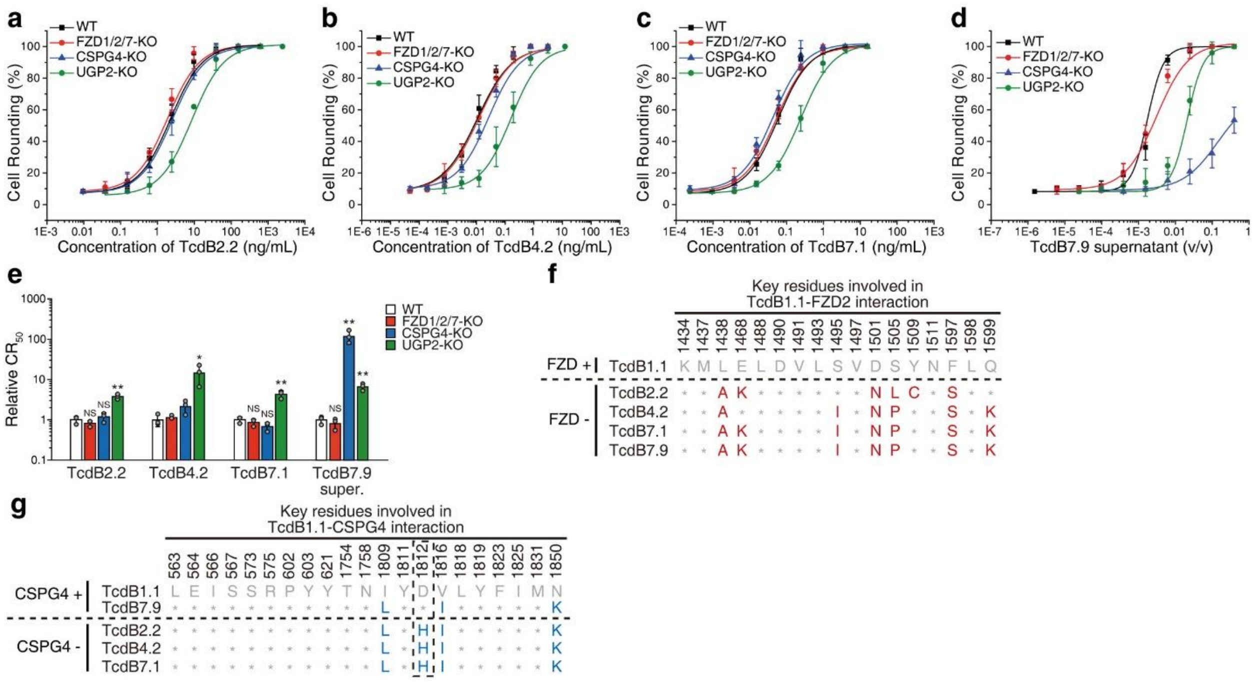

Supplementary Figure 4: TcdB subtypes show variable dependency on FZD and CSPG4 receptors.

a-e HeLa-WT, FZD1/2/7-KO, CSPG4-KO, and UGP2-KO cells were exposed to either recombinant TcdB2.2 (**a**), TcdB4.2 (**b**), TcdB7.1 (**c**), or culture supernatant from native *C. difficile* strain expressing TcdB7.9 (**d**) for 24 h. The percentages of round-shaped cells were plotted over toxin concentrations or supernatant dilutions. The relative CR_{50} values in different cell lines were normalized to the WT and plotted as bar-chart (**e**). Error bars indicate mean ± s.d.; $N = 3$ (biologically independent experiments); *, $p < 0.05$; **, $p < 0.01$; NS, not significant (Student's *t*-test, two-sided).

f A list of residues across tested TcdB subtypes at 17 key positions mediating TcdB1.1-FZD2 interactions. These positions are based on the crystal structure of TcdB-FZD complex (PDB: 6C0B).

g A list of residues across tested TcdB subtypes at 21 key positions mediating TcdB1.1-CSPG4 interactions. These positions are based on the cryo-EM structure of TcdB-CSPG4 complex (PDB: 7ML7). Residue 1812 was highlighted with a dash box.

Source data are provided as a Source Data file.

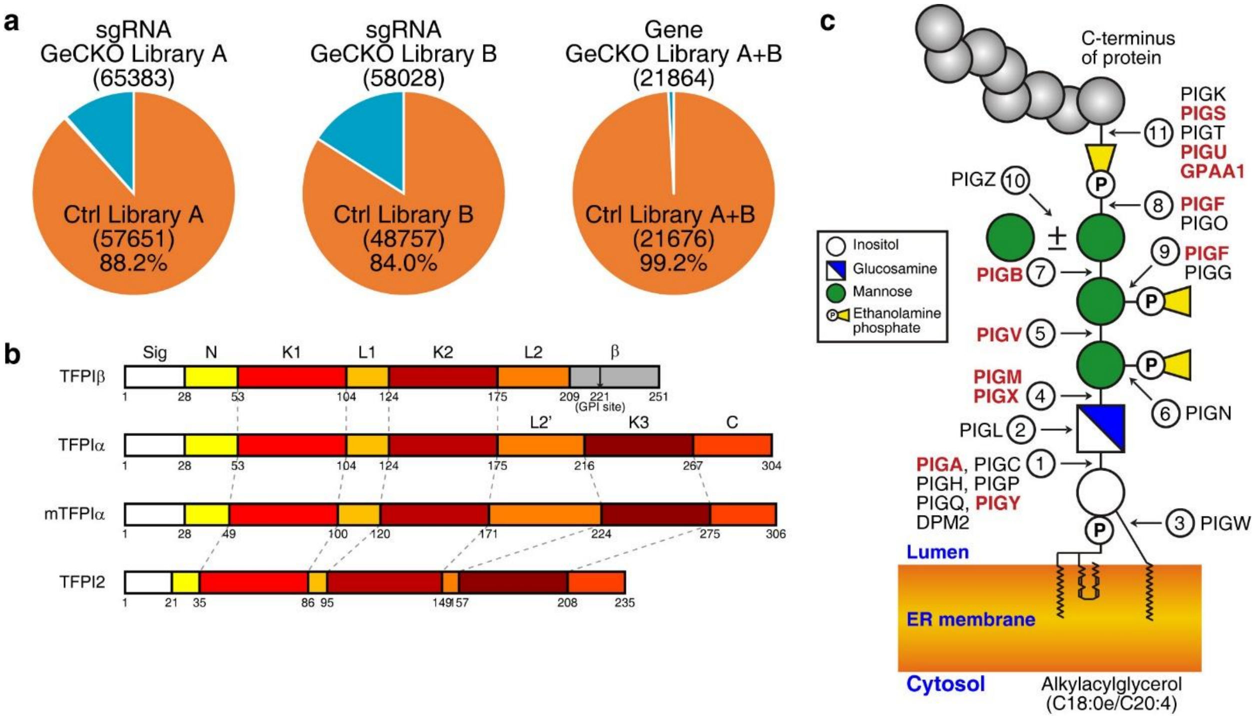

Supplementary Figure 5: CRISPR-Cas9-mediated genome-wide screen for TcdB4.2.

a Recovery rates of sgRNAs and genes identified in the control (Ctrl) cell libraries compared with the original GeCKO-V2 libraries.

b Schematic diagrams of TFPIβ (GPI-anchored form), TFPIα (secreted form), mouse TFPIα (secreted form), and TFPI2 structures. The numbers indicate the position of amino acid residues. Sig, signal peptide; N, N-terminal domain; K1, BPTI/Kunitz inhibitor domain 1; L1, loop 1; K2, BPTI/Kunitz inhibitor domain 2; L2, loop 2; β, GPI anchor sequence for TFPIβ; K3, BPTI/Kunitz inhibitor domain 3; C, C-terminal domain. The arrow indicates the GPI modification site in TFPIβ.

c Schematic diagrams of GPI architecture and the enzymes involved in each step of the GPI biosynthesis pathway. The genes identified in the TcdB4.2 screen are highlighted in red.

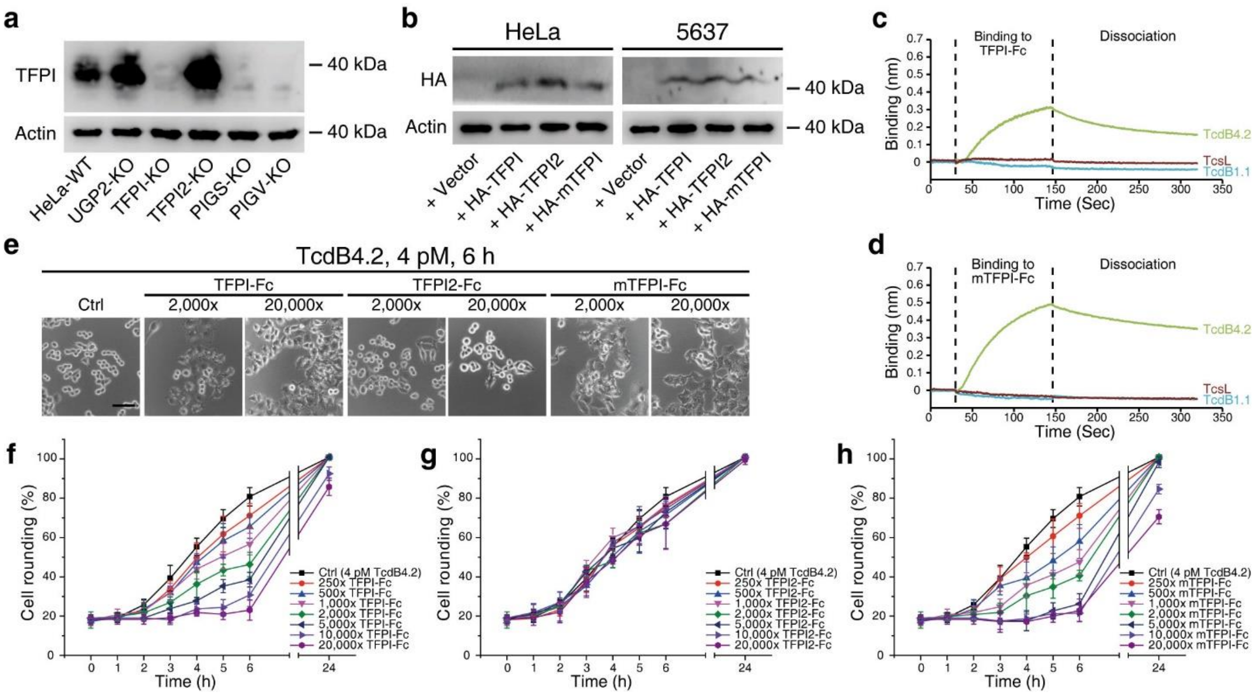

Supplementary Figure 6: TFPI is a receptor for TcdB4.2.

a The expression levels of TFPI in HeLa-WT, UGP2-KO, TFPI-KO, TFPI2-KO, PIGS-KO, and PIGV-KO cells were examined using immunoblot detecting endogenous TFPI. Actin was used as a loading control. Representative images were shown from two independent experiments.

b TFPI, TFPI2, and mTFPI were expressed in HeLa or 5637 cells via lentiviral transduction. Expressed exogenous TFPI proteins in cells were confirmed via immunoblot detecting the triple HA tag fused to their N-termini. Actin was used as a loading control. Representative images were shown from two independent experiments.

c-d Binding of 500 nM TcdB1.1, TcdB4.2, and TcsL to Fc-tagged TFPI (**c**) and mTFPI (**d**) was examined using BLI assays. Representative sensorgrams from one of three independent experiments are shown.

e-h HeLa cells were exposed to either TcdB4.2 alone (4 pM) or TcdB4.2 pre-incubated with Fc-tagged TFPI (**f**), TFPI2 (**g**), or mTFPI (**h**) at the indicated molar ratios (1:250 ~ 1:20,000) on ice for 1 h. Representative images of the cell rounding effect at indicated conditions are shown (**e**). Scale bar, 20 µm. The percentages of cell rounding over time were recorded. Error bars indicate mean ± s.d.; $N = 3$ (biologically independent experiments).

Source data are provided as a Source Data file.

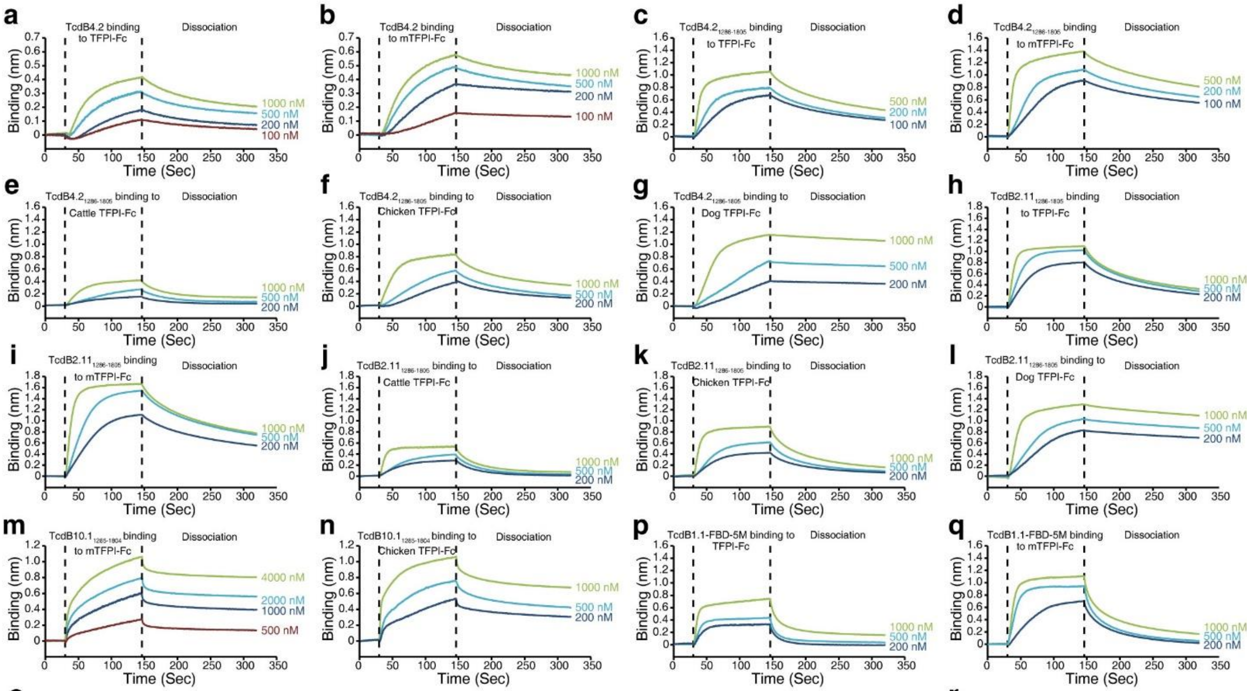

o

Toxin	TFPI-Fc	k_a ($M^{-1}s^{-1}$)	k_d (s^{-1})	K_D (M)
**TcdB4.2**	Human	$2.00 \times 10^4 \pm 1.33 \times 10^3$	$4.47 \times 10^{-3} \pm 1.62 \times 10^{-4}$	$2.27 \times 10^{-7} \pm 2.28 \times 10^{-8}$
	Mouse	$2.31 \times 10^4 \pm 1.03 \times 10^3$	$1.44 \times 10^{-3} \pm 7.89 \times 10^{-5}$	$6.25 \times 10^{-8} \pm 6.20 \times 10^{-9}$
**TcdB4.2$_{1286\text{-}1805}$**	Human	$1.43 \times 10^5 \pm 2.19 \times 10^3$	$7.08 \times 10^{-3} \pm 8.77 \times 10^{-5}$	$4.95 \times 10^{-8} \pm 1.37 \times 10^{-9}$
	Mouse	$1.43 \times 10^5 \pm 2.32 \times 10^3$	$3.44 \times 10^{-3} \pm 7.46 \times 10^{-5}$	$2.41 \times 10^{-8} \pm 9.13 \times 10^{-10}$
	Cattle	$2.57 \times 10^4 \pm 8.42 \times 10^2$	$1.38 \times 10^{-2} \pm 2.00 \times 10^{-4}$	$5.36 \times 10^{-7} \pm 2.77 \times 10^{-8}$
	Chicken	$2.63 \times 10^4 \pm 8.24 \times 10^2$	$8.85 \times 10^{-3} \pm 1.70 \times 10^{-4}$	$3.36 \times 10^{-7} \pm 1.70 \times 10^{-8}$
	Dog	$2.60 \times 10^4 \pm 3.82 \times 10^2$	$3.95 \times 10^{-4} \pm 2.56 \times 10^{-5}$	$1.52 \times 10^{-8} \pm 1.21 \times 10^{-9}$
**TcdB2.11$_{1286\text{-}1805}$**	Human	$8.25 \times 10^4 \pm 1.33 \times 10^3$	$1.09 \times 10^{-2} \pm 1.12 \times 10^{-4}$	$1.33 \times 10^{-7} \pm 3.49 \times 10^{-9}$
	Mouse	$6.86 \times 10^4 \pm 1.30 \times 10^3$	$6.23 \times 10^{-3} \pm 1.07 \times 10^{-4}$	$9.09 \times 10^{-8} \pm 3.29 \times 10^{-9}$
	Cattle	$7.76 \times 10^4 \pm 1.74 \times 10^3$	$2.40 \times 10^{-2} \pm 2.09 \times 10^{-4}$	$3.10 \times 10^{-7} \pm 9.63 \times 10^{-9}$
	Chicken	$8.02 \times 10^4 \pm 1.22 \times 10^3$	$1.65 \times 10^{-2} \pm 1.18 \times 10^{-4}$	$2.05 \times 10^{-7} \pm 4.60 \times 10^{-9}$
	Dog	$5.67 \times 10^4 \pm 5.62 \times 10^2$	$1.19 \times 10^{-3} \pm 2.20 \times 10^{-5}$	$2.09 \times 10^{-8} \pm 5.95 \times 10^{-10}$
**TcdB10.1$_{1285\text{-}1804}$**	Mouse	$1.37 \times 10^4 \pm 5.75 \times 10^2$	$6.37 \times 10^{-3} \pm 1.50 \times 10^{-4}$	$4.65 \times 10^{-7} \pm 3.05 \times 10^{-8}$
	Chicken	$8.52 \times 10^4 \pm 1.48 \times 10^3$	$6.00 \times 10^{-3} \pm 1.10 \times 10^{-4}$	$7.05 \times 10^{-8} \pm 2.51 \times 10^{-9}$

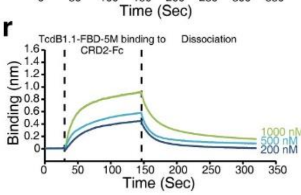

s

Toxin	Receptor	k_a ($M^{-1}s^{-1}$)	k_d (s^{-1})	K_D (M)
**TcdB1.1-FBD-5M**	TFPI	$1.20 \times 10^5 \pm 4.25 \times 10^3$	$4.19 \times 10^{-2} \pm 7.05 \times 10^{-4}$	$3.49 \times 10^{-7} \pm 4.47 \times 10^{-8}$
	mTFPI	$7.64 \times 10^4 \pm 3.13 \times 10^3$	$2.08 \times 10^{-2} \pm 2.65 \times 10^{-4}$	$2.72 \times 10^{-7} \pm 1.10 \times 10^{-8}$
	CRD2	$3.00 \times 10^4 \pm 1.26 \times 10^3$	$2.41 \times 10^{-2} \pm 3.38 \times 10^{-4}$	$8.01 \times 10^{-7} \pm 4.47 \times 10^{-8}$

Supplementary Figure 7: Quantification of TcdB-TFPI interactions using BLI assays.

a-b Binding kinetics and affinity were determined using BLI assays for interactions between full-length TcdB4.2 and TFPI-Fc (**a**) or mTFPI-Fc (**b**). Representative sensorgrams from one of two independent experiments are shown.

c-g Binding kinetics and affinity were determined using BLI assays for interactions between TcdB4.2$_{1286\text{-}1805}$ and TFPI-Fc (**c**), mTFPI-Fc (**d**), cattle TFPI-Fc (**e**), chicken TFPI-Fc (**f**), and dog TFPI-Fc (**g**). Representative sensorgrams from one of two independent experiments are shown.

(h-l) Binding kinetics and affinity were determined using BLI assays for interactions between TcdB2.11$_{1286\text{-}1805}$ and TFPI-Fc (**h**), mTFPI-Fc (**i**), cattle TFPI-Fc (**j**), chicken TFPI-Fc (**k**), and dog TFPI-Fc (**l**). Representative sensorgrams from one of two independent experiments are shown.

(m-n) Binding kinetics and affinity were determined using BLI assays for interactions between TcdB10.1$_{1285\text{-}1804}$ and mTFPI-Fc (**m**), and chicken TFPI-Fc (**n**). Representative sensorgrams from one of two independent experiments are shown.

o Summary of the binding kinetics between TcdB variants and TFPI across several species (mean ± s.d.).

p-r Binding kinetics and affinity were determined using BLI assays for interactions between TcdB1.1-FBD-5M and TFPI-Fc (**p**), mTFPI-Fc (**q**), and FZD-CRD2-Fc (**r**). Representative sensorgrams from one of two independent experiments are shown.

s Summary of the binding kinetics between TcdB1.1-FBD-5M and the indicated proteins (mean ± s.d.).

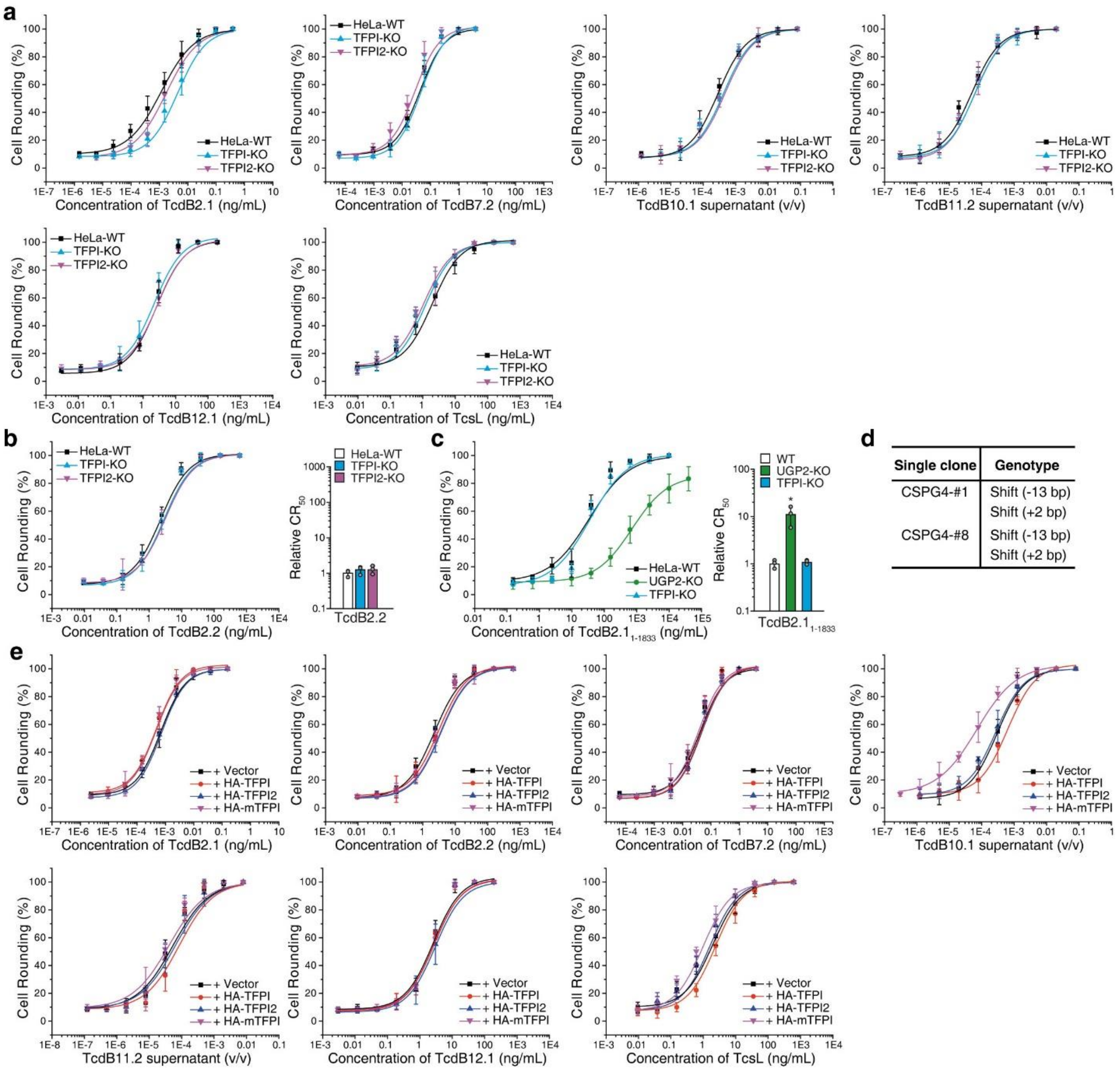

Supplementary Figure 8: Investigating TFPI dependency for TcdB variants that do not recognize FZDs.

a HeLa-WT, TFPI-KO, and TFPI2-KO cells were exposed to recombinant TcdB2.1, TcdB7.2, TcsL, or culture supernatants of *C. difficile* strains expressingTcdB10.1, TcdB11.2, or TcdB12.1 for 24 h. The percentages of round-shaped cells were plotted over toxin concentrations or supernatant dilutions. Error bars indicate mean ± s.d.; N = 3 (biologically independent experiments).

b HeLa-WT, TFPI-KO, and TFPI2-KO cells were exposed to recombinant TcdB2.2 for 24 h. The percentages of round-shaped cells were plotted over toxin concentrations. The relative CR_{50} values in different cell lines were normalized to the WT and plotted as bar-chart (right panel). Error bars indicate mean ± s.d.; N = 3 (biologically independent experiments).

c HeLa-WT, UGP2-KO, and TFPI-KO cells were exposed to recombinant TcdB2.1$_{1-1833}$ for 24 h. The percentages of round-shaped cells were plotted over toxin concentrations. The relative CR_{50} values in different cell lines were normalized to the WT and plotted as bar-chart (right panel). Error bars indicate mean ± s.d.; $N = 3$ (biologically independent experiments); *, $p < 0.05$ (Student's *t*-test, two-sided).

d Genotypes of CSPG4-KO single clones.

e HeLa cells overexpressing HA-tagged TFPI, TFPI2, or mTFPI via lentiviral transduction were exposed to recombinant TcdB2.1, TcdB2.2, TcdB7.2, TcsL, or culture supernatants of *C. difficile* strains expressingTcdB10.1, TcdB11.2, or TcdB12.1, for 24 h. The percentages of round-shaped cells were plotted over toxin concentrations or supernatant dilutions. Error bars indicate mean ± s.d.; $N = 3$ (biologically independent experiments).

Source data are provided as a Source Data file.

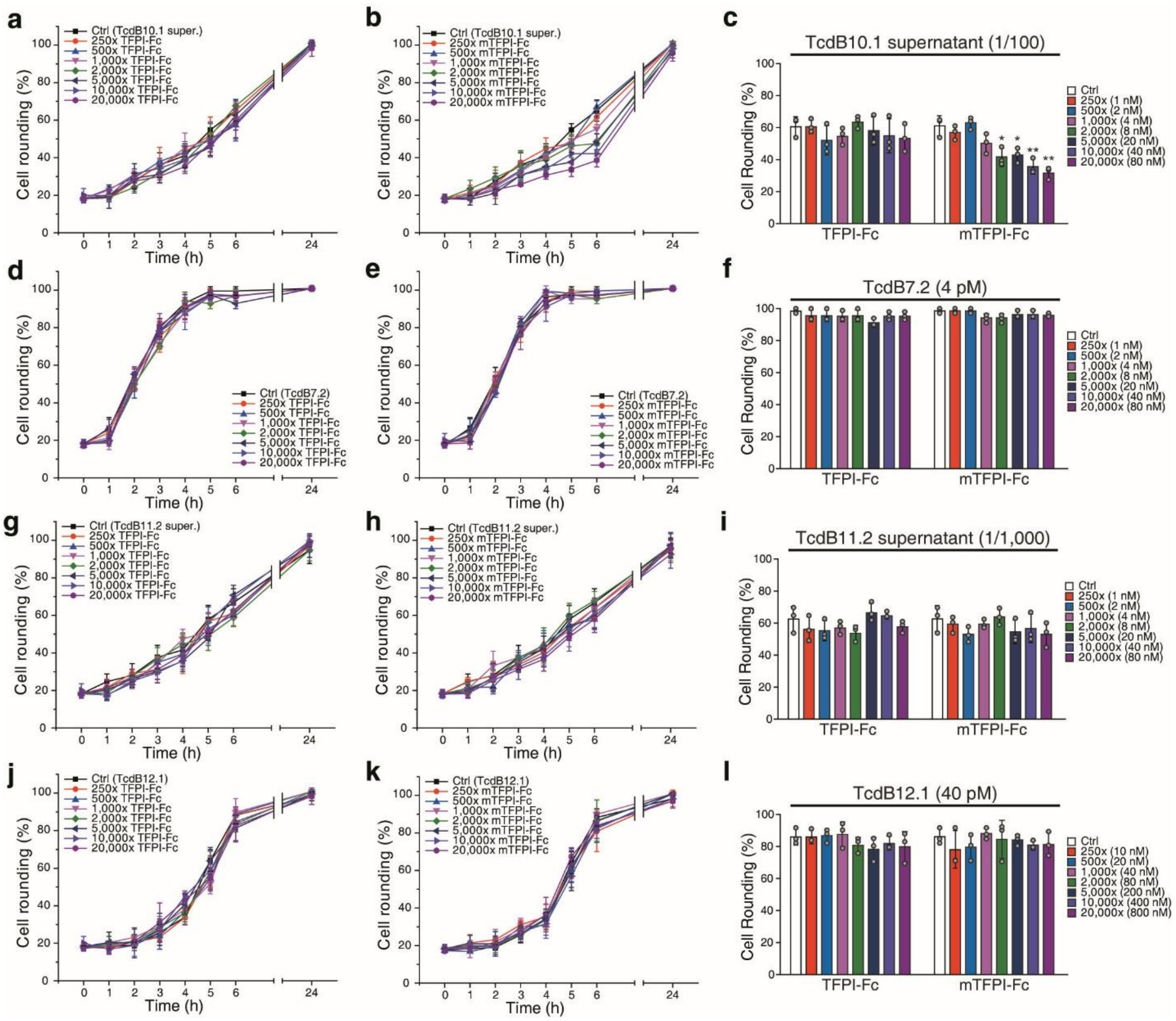

Supplementary Figure 9: TcdB10.1 utilizes mouse TFPI as receptor.

HeLa cells were exposed to the culture supernatants of *C. difficile* strains expressing TcdB10.1 (**a-c**), the recombinant TcdB7.2 (**d-f**), or the culture supernatants of *C. difficile* strains expressing TcdB11.2 (**g-i**), or TcdB12.1 (**j-l**) with or without preincubation with Fc-tagged TFPI (**a, d, g, j**) or mTFPI (**b, e, h, k**) at the indicated ratio on ice for 1 h. The percentages of cell rounding were recorded over time. The percentages of cell-rounding at 6 h incubation were plotted as bar-charts (**c, f, i, l**). Error bars indicate mean ± s.d.; $N = 3$ (biologically independent experiments); *, $p < 0.05$; **, $p < 0.01$ (Student's *t*-test, two-sided).

Source data are provided as a Source Data file.

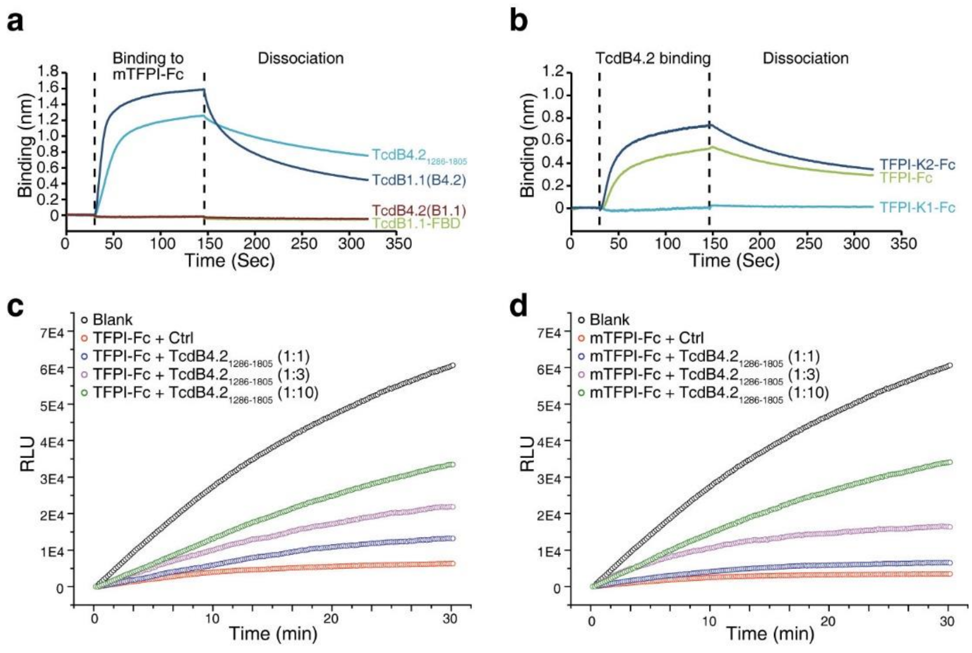

Supplementary Figure10: Characterization of TcdB4.2-TFPI interactions.

a Binding of 500 nM TcdB1.1-FBD, TcdB4.2$_{1286\text{-}1805}$, TcdB4.2(B1.1), and TcdB1.1(B4.2) to Fc-tagged mTFPI was examined using BLI assays. Representative sensorgrams from one of three independent experiments are shown.

b Binding of 500 nM TcdB4.2 full-length toxin to Fc-tagged TFPI, TFPI-K1, and TFPI-K2 was examined using BLI assays. Representative sensorgrams from one of three independent experiments are shown.

c-d FXa's enzymatic activity can be inhibited by Fc-tagged TFPI (**c**, 5 ng/mL) or mTFPI (**d**, 5 ng/mL). The inhibitory effect of TFPI can be blocked by adding TcdB4.2$_{1286\text{-}1805}$ in a dose-dependent manner (1:1, 1:3, or 1:10 molar ratio). Representative curves from one of three independent experiments are shown.

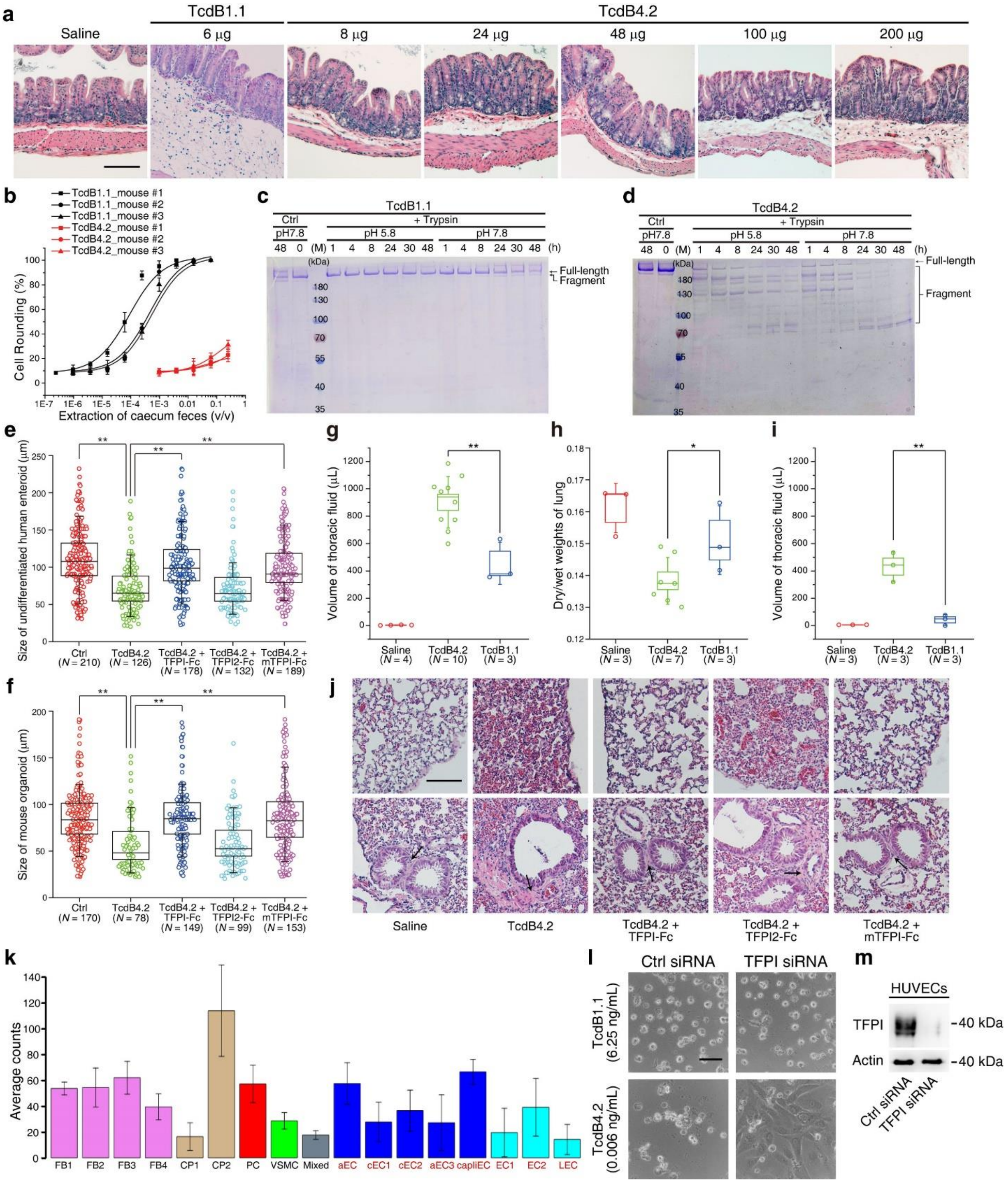

Supplementary Figure 11: TFPI-Fc protein blocks the in vivo toxicity of TcdB4.2.

a TcdB1.1 or TcdB4.2 was injected into the cecum of mice at indicated doses for 6 h. Saline was injected as control in parallel. The cecum was harvested and processed with hematoxylin and eosin staining. Scale bar, 100 μm. Representative images from one of two independent experiments.

b 8 µg TcdB1.1 or TcdB4.2 was injected into the cecum of mice (3 mice per group) for 6 h. Luminal contents were extracted with 1 mL PBS. The extractions were filtered and incubated with HeLa cells for 24 h. The percentages of round-shaped cells were plotted over dilutions. Error bars indicate mean ± s.d., $N = 3$ (biologically independent experiments).

c-d Limited trypsin digestion was performed on TcdB1.1 and TcdB4.2 at pH 5.8 and pH 7.8. Shown are representative SDS–PAGE gels with Coomassie blue staining from two independent experiments.

e-f Cultured undifferentiated human enteroids (**e**) and mouse intestinal organoids (**f**) were exposed to either TcdB4.2 alone (10 pM) or TcdB4.2 pre-incubated with Fc-tagged TFPI, TFPI2, or mTFPI (100 nM) for 8 h. PBS was used as control (Ctrl). The range of boxes indicates ± s.e.m.; whiskers indicate ± s.d.; percentiles indicate median; **, $p < 0.01$ (Student's *t*-test, two-sided).

g-h The same amount of TcdB4.2 or TcdB1.1 (50 ng per 25 g bodyweight) was injected intraperitoneally into mice and the lung tissues were harvested and analyzed 15 h later. The volume of fluid in the thoracic cavity (**g**) and the dry-to-wet weight ratios of lung tissues (**h**) were shown. Injection of saline was included as a control. The range of boxes indicates ± s.e.m.; whiskers indicate ± s.d.; percentiles indicate median; *, $p < 0.05$; **, $p < 0.01$ (Student's *t*-test, two-sided).

i Experiments were carried out as described in panel **g**, except that the lung tissues were harvested 4 h after injection. Injection of saline was included as a control. The range of boxes indicates ± s.e.m.; whiskers indicate ± s.d.; percentiles indicate median; **, $p < 0.01$ (Student's *t*-test, two-sided).

j Experiments were carried out as described in Fig. 4e-g, and the indicated lung tissues were harvested and subjected to histological analysis (H&E staining). Alveolar hemorrhage (upper panels) and widening of perivascular space (arrows in lower panels) were observed. These pathological changes were smaller in the TcdB4.2 + TFPI-Fc and TcdB4.2 + mTFPI-Fc groups. Scale bar, 200 µm.

k Expression of TFPI in various lung tissue cells were plotted based on published single cell RNAseq data (http://betsholtzlab.org/VascularSingleCells/database.html)[79,80]. FB: Vascular fibroblast-like cells; CP: Cartilage perichondrium; PC: Pericytes; VSMC: Vascular smooth muscle cells; EC: Endothelial cells (highlighted in red); capil - capillary; a - arterial; c - continuum; L - Lymphatic; 1,2,3,4 - subtypes.

l Experiments were carried out as described in Fig. 4h-i. Representative images of the cell rounding effect in HUVECs are shown. Scale bar, 20 µm.

m The TFPI-targeting or non-targeting control siRNAs were transfected into HUVECs. Cell lysates were analyzed by immunoblot detecting TFPI. Actin served as a loading control. Representative images were shown from two independent experiments.

Source data are provided as a Source Data file.

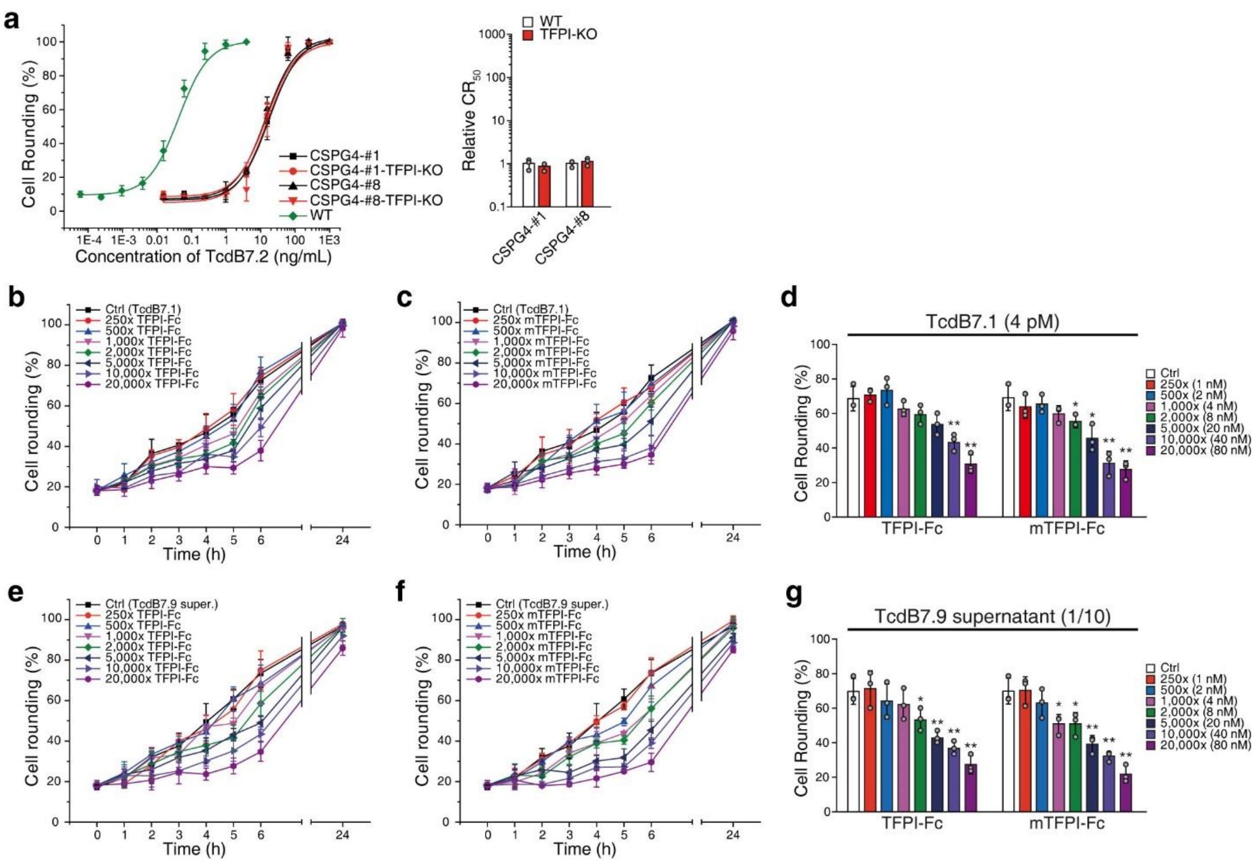

Supplementary Figure 12: TFPI is a receptor for TcdB7.1 and TcdB7.9 but not TcdB7.2.

a HeLa-WT, two CSPG4 KO single clones (CSPG4#1 and CSPG4#8), and two CSPG4/TFPI double cells (CSPG4#1-TFPI-KO and CSPG4#8-TFPI-KO) were exposed to TcdB7.2 for 24 h. The percentages of rounded cells were plotted over toxin concentrations. Their relative CR_{50} values are plotted in a bar-chart (right panel). Error bars indicate mean ± s.d.; $N = 3$ (biologically independent experiments).

b-g HeLa cells were exposed to recombinant TcdB7.1 (**b-d**) or the culture supernatant of *C. difficile* strain expressing TcdB7.9 (**e-g**), with or without preincubation with Fc-tagged TFPI (**b, e**) or mTFPI (**c, f**) at the indicated ratio on ice for 1 h. The percentages of cell rounding were recorded over time. The percentage of cell-rounding at 6 h incubation was plotted as a bar-chart (**d, g**). Error bars indicate mean ± s.d.; $N = 3$ (biologically independent experiments); *, $p < 0.05$; **, $p < 0.01$ (Student's *t*-test, two-sided).

Source data are provided as a Source Data file.

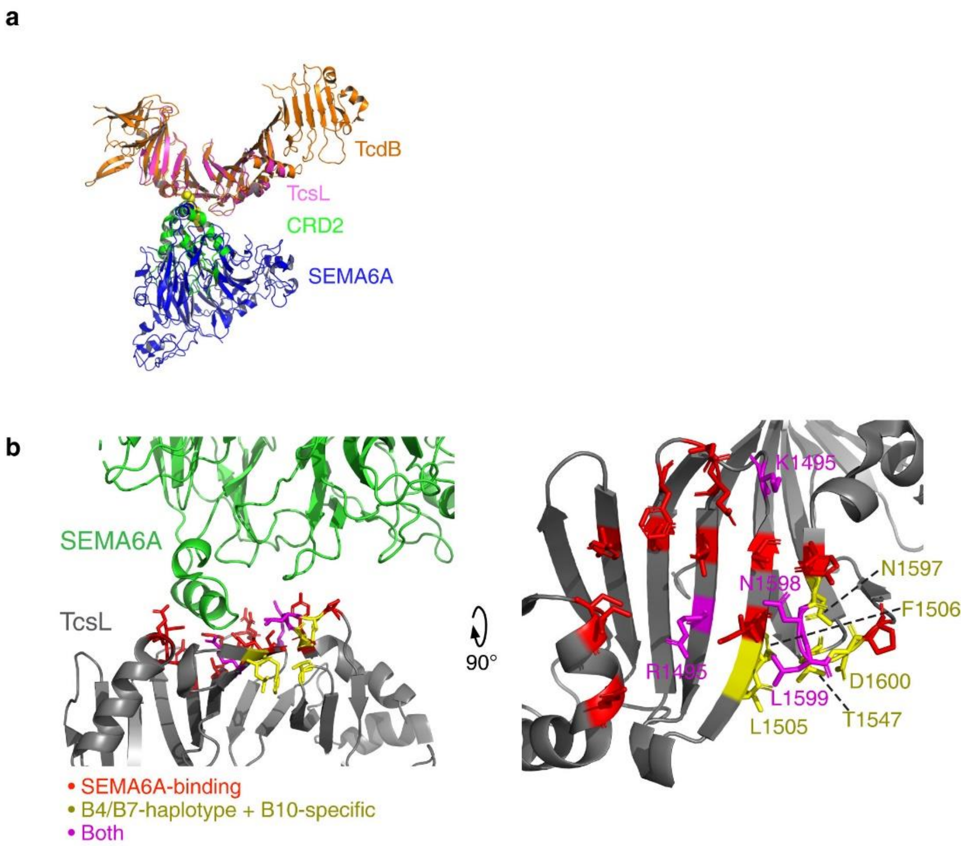

Supplementary Figure 13: TFPI-binding site is at a location similar to the SEMA6A-binding site in TcsL.

a Structures of the TcsL-SEMA6A complex (PDB: 6WTS)[39] and the TcdB1-CRD2 complex (PDB: 6C0B) were superimposed based on the DRBD (R.M.S.D = 1.216 Å). TcdB, TcsL, CRD2, and SEMA6A were shown as cartoons and colored in orange, pink, green, and blue, respectively. PAM was shown as spheres and colored yellow.

b The interface of TcsL-SEMA6A is shown (PDB: 6WTS), with SEMA6A-binding residues colored in red. Residues in TcsL corresponding to key residues for TFPI-binding (B4/B7-haplotype and B10-specific substitutions) are colored in yellow and overlapping (common) residues colored in pink.

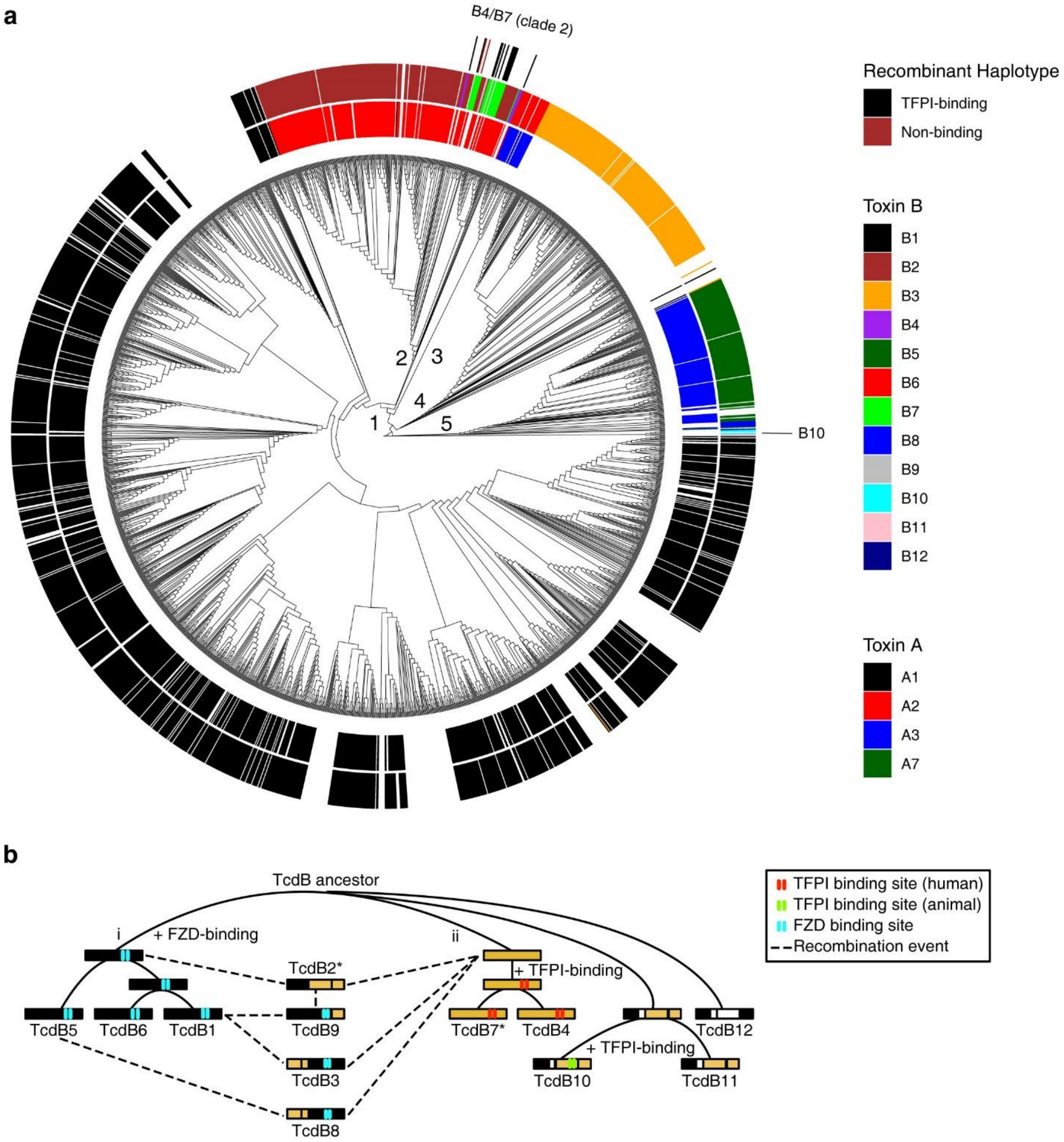

Supplementary Figure 14. An evolutionary pathway for TcdB subtypes.

a Phylogenomic tree of 2,118 representative *C. difficile* genomes from the NCBI database derived from our previous study[50], highlighting sub-lineages within clade 2 that contain the TFPI-binding B4/B7 haplotype (black bars on outer ring). Also shown are two genomes with TcdB7 sequences that lack the B4/B7 haplotype and do not bind TFPI (brown bars). Not all toxin subtypes are represented as this tree is based only on high-quality genomes passing coverage and alignment quality thresholds.

b Model for the evolution of TcdB subtypes. An ancestral TcdB toxin diverged into several lineages including the major lineages i and ii. FZD binding emerged in lineage i, while TFPI binding emerged in lineage ii leading to B4/B7. Independently, through separate substitutions, non-human TFPI binding emerged in the lineage leading to TcdB10.

Full scans for immunoblots
Supplementary Figure 6a (upper panel)

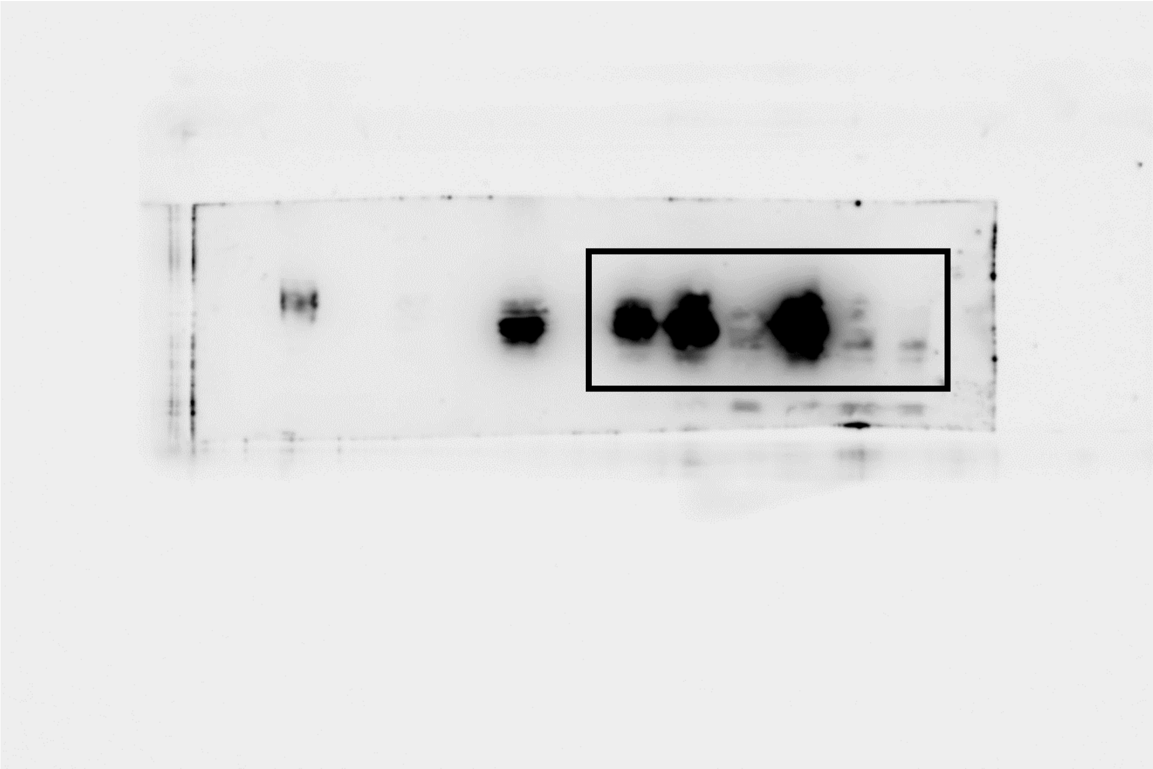

Supplementary Figure 6a (lower panel)

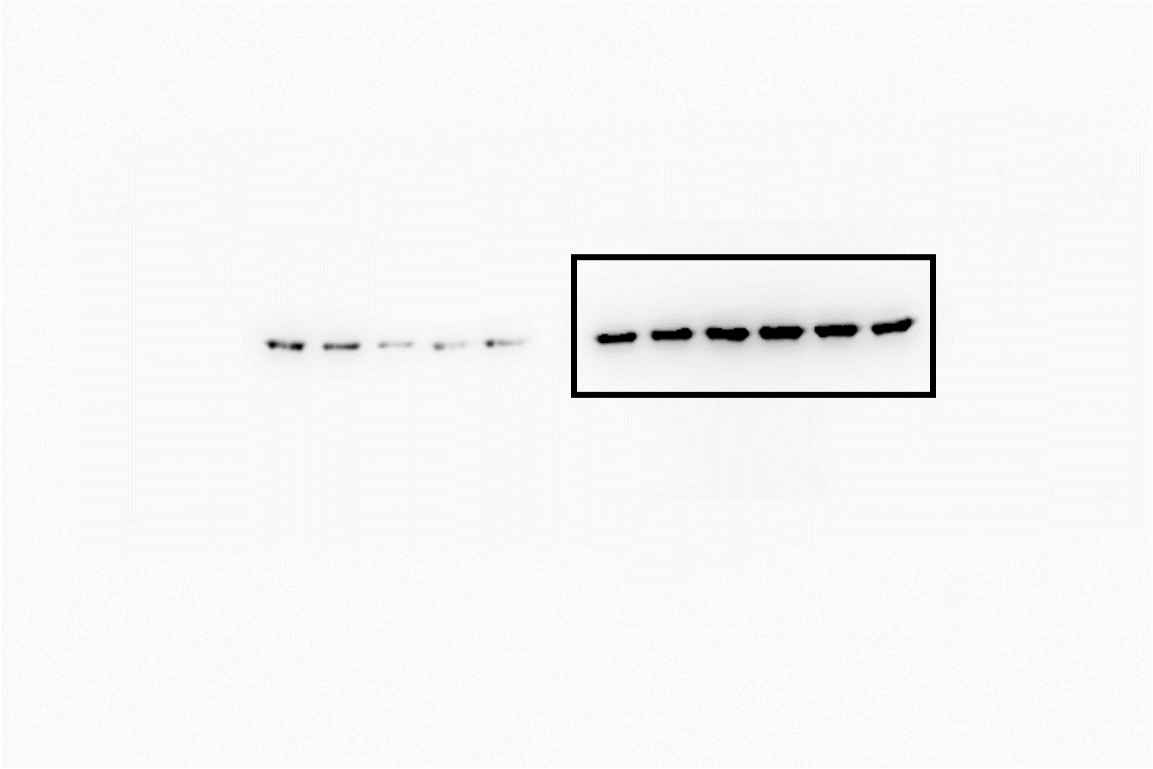

Supplementary Figure 6b (upper panel)

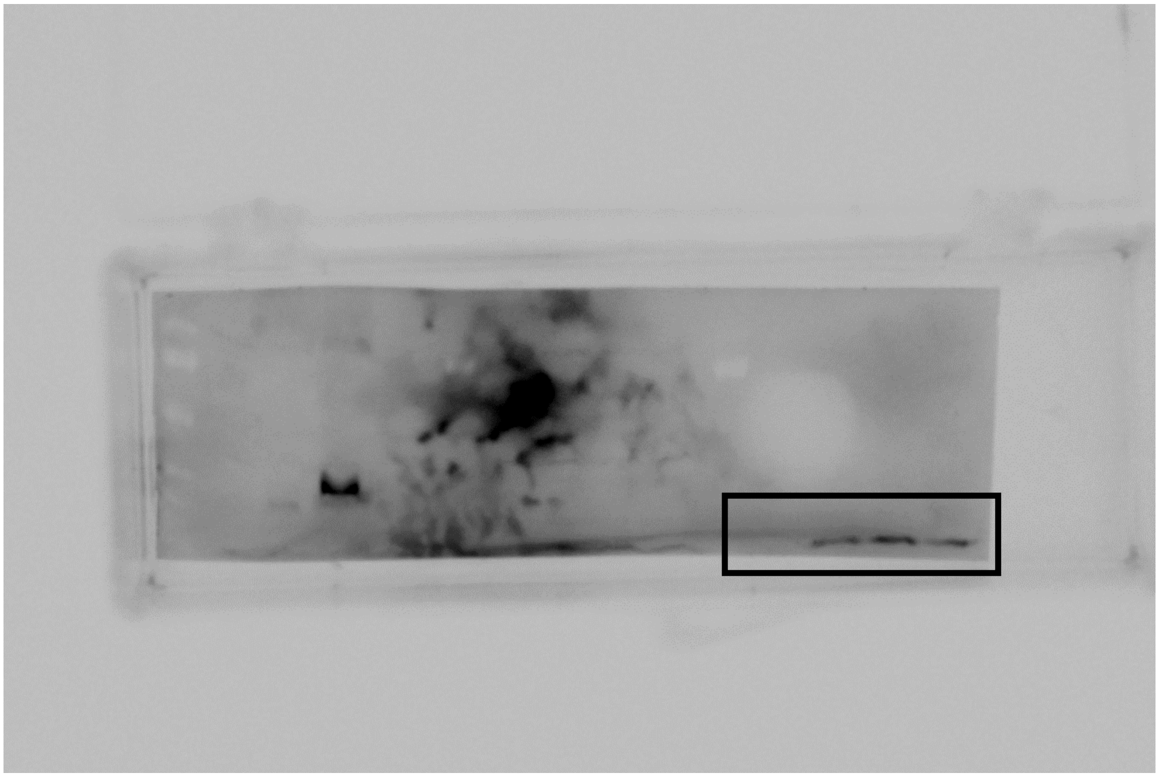

Supplementary Figure 6b (upper panel)

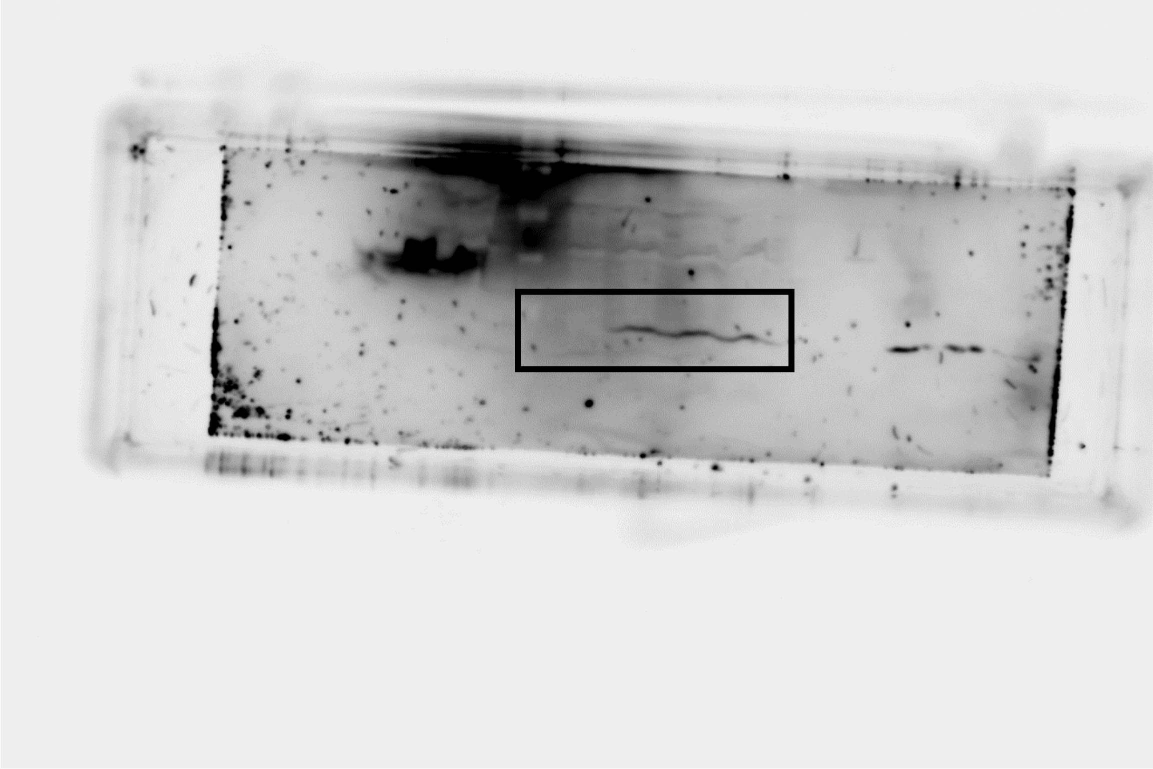

Supplementary Figure 6b (lower panel)

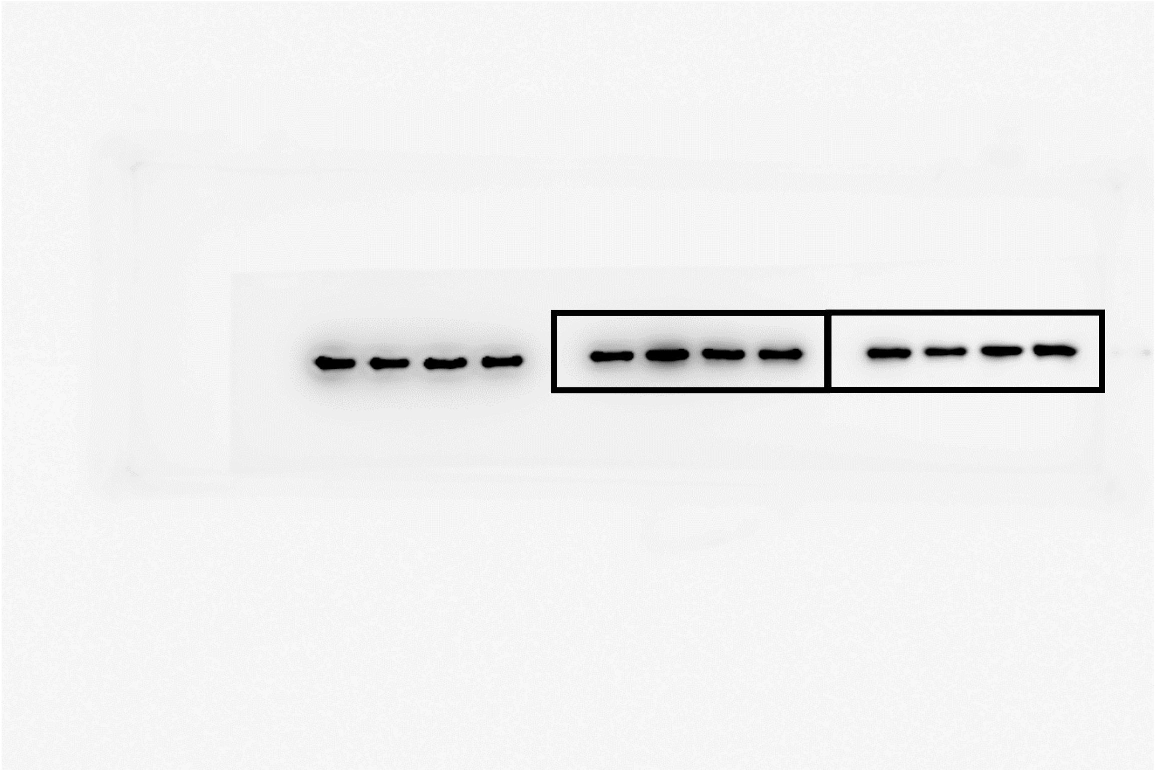

Supplementary Figure 11m (upper panel)

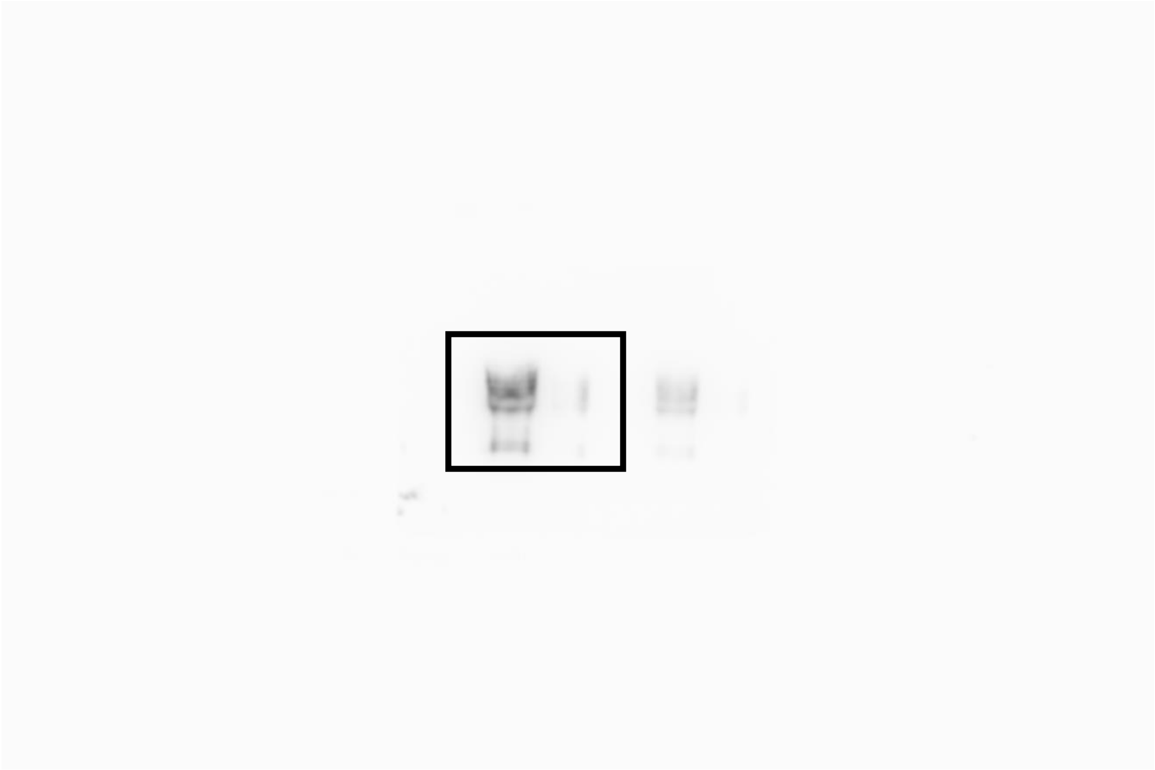

Supplementary Figure 11m (lower panel)

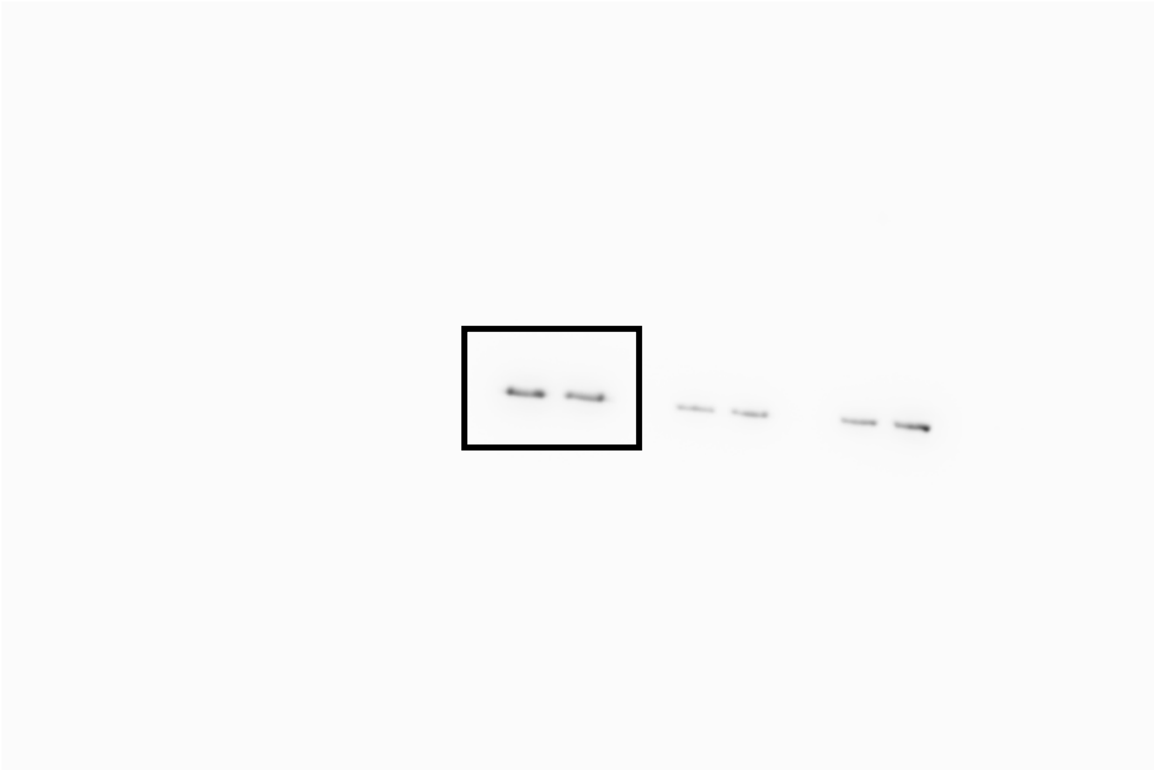

Supplementary Table 1: Information on *C. difficile* strains used in this study.

Strain name	TcdA/TcdB subtype	Accession	Source	References
CD128	A2 (putative)/B2.2	SAMN10766005	Brigham and Women's Hospital	[1]
CD38	A2/B2.22	SAMN10766054	Brigham and Women's Hospital	[1]
CD02-20171122	-/B3.1	SAMN10715303	Brigham and Women's Hospital	[1]
CD82-20180223	A2 (putative)/B4.2	SAMN08885860	Brigham and Women's Hospital	[1]
CD55-20181128	A3.1/B5.1	SAMN08784007	Brigham and Women's Hospital	[1]
DSM102860	A3.2/B6.1	SAMN06619923	DSMZ-German Collection of Microorganisms and Cell Cultures GmbH	[2]
CD16	A2.2/B7.1	SAMN07974944	Brigham and Women's Hospital	[1]
CD79-20180223	A2.6/B7.2	SAMN08885857	Brigham and Women's Hospital	[1]
LIBA-7678	-/B7.9	[7]	LIBA, UCR	[3]
V1787-20180424	-/B8.3	SAMN09060514	Brigham and Women's Hospital	[1]
CD61-20180126	A2.5/B9.1	SAMN08784013	Brigham and Women's Hospital	[1]

CD10-165	-/B10.1	SAMN03015184	Villefranche-sur-Saône Hospital	[4]
HMX-149	-/B11.2	[8]	LIBA, UCR	[5]
C. diff 173070	-/B12.1	SAMEA104432225	University Hospital Muenster	[6]

Supplementary Table 1 References

[1] Worley J, Delaney ML, Cummins CK, DuBois A, Klompas M, Bry L. Genomic Determination of Relative Risks for *Clostridioides difficile* Infection From Asymptomatic Carriage in Intensive Care Unit Patients. Clin Infect Dis. 2021 Oct 5;73(7):e1727-e1736. doi: 10.1093/cid/ciaa894. PMID: 32676661.

[2] Riedel T, Wetzel D, Hofmann JD, Plorin SPEO, Dannheim H, Berges M, Zimmermann O, Bunk B, Schober I, Spröer C, Liesegang H, Jahn D, Overmann J, Groß U, Neumann-Schaal M. High metabolic versatility of different toxigenic and non-toxigenic *Clostridioides difficile* isolates. Int J Med Microbiol. 2017 Sep;307(6):311-320. doi: 10.1016/j.ijmm.2017.05.007. Epub 2017 Jun 1. PMID: 28619474.

[3] Ramírez-Vargas G, Rodríguez C. Putative Conjugative Plasmids with tcdB and cdtAB Genes in *Clostridioides difficile*. Emerg Infect Dis. 2020 Sep;26(9):2287-2290. doi: 10.3201/eid2609.191447. PMID: 32818425

[4] Monot M, Eckert C, Lemire A, Hamiot A, Dubois T, Tessier C, Dumoulard B, Hamel B, Petit A, Lalande V, Ma L, Bouchier C, Barbut F, Dupuy B. *Clostridium difficile*: New Insights into the Evolution of the Pathogenicity Locus. Sci Rep. 2015 Oct 8;5:15023. doi: 10.1038/srep15023. PMID: 26446480; PMCID: PMC4597214.

[5] Ramírez-Vargas G, López-Ureña D, Badilla A, Orozco-Aguilar J, Murillo T, Rojas P, Riedel T, Overmann J, González G, Chaves-Olarte E, Quesada-Gómez C, Rodríguez C. Novel Clade C-I *Clostridium difficile* strains escape diagnostic tests, differ in pathogenicity potential and carry toxins on extrachromosomal elements. Sci Rep. 2018 Sep 17;8(1):13951. doi: 10.1038/s41598-018-32390-6. PMID: 30224751; PMCID: PMC6141592.

[6] Janezic S, Marín M, Martín A, Rupnik M. A new type of toxin A-negative, toxin B-positive *Clostridium difficile* strain lacking a complete tcdA gene. J Clin Microbiol. 2015 Feb;53(2):692-5. doi: 10.1128/JCM.02211-14. Epub 2014 Nov 26. PMID: 25428159; PMCID: PMC4298534.

[7] https://microbesng.com/portal/projects/FB43968C-E9EF-4270-9D1A-054457CC9B54/

[8] https://microbesng.com/portal/projects/149D9096-03DF-4C6B-BF5F-2119D1AE7B68/
