# Supplementary material for: Identification of TFPI as a receptor reveals recombination-driven receptor switching in Clostridioides difficile toxin B variants
Source: Nat Commun. 2022 Nov 9;13:6786. doi: 10.1038/s41467-022-33964-9 (PMC9646764; doi:10.1038/s41467-022-33964-9)
Supplement: Supplementary file 2 — Description of Additional Supplementary Files [file 41467_2022_33964_MOESM2_ESM.pdf]

Description of Additional Supplementary Files:

Supplementary Data1: Excel file listing CRISPR-Cas9 screening results.
