# Supplementary material for: Identification of TFPI as a receptor reveals recombination-driven receptor switching in Clostridioides difficile toxin B variants
Source: Nat Commun. 2022 Nov 9;13:6786. doi: 10.1038/s41467-022-33964-9 (PMC9646764; doi:10.1038/s41467-022-33964-9)
Supplement: Supplementary file 4 — Reporting Summary [file 41467_2022_33964_MOESM4_ESM.pdf]

nature portfolio

Corresponding author(s): Min Dong, Songhai Tian, Andrew Doxey

Last updated by author(s): Sep 29, 2022

Reporting Summary

Nature Portfolio wishes to improve the reproducibility of the work that we publish. This form provides structure for consistency and transparency in reporting. For further information on Nature Portfolio policies, see our Editorial Policies and the Editorial Policy Checklist.

Statistics

For all statistical analyses, confirm that the following items are present in the figure legend, table legend, main text, or Methods section.

n/a	Confirmed	
☐	☒	The exact sample size (*n*) for each experimental group/condition, given as a discrete number and unit of measurement
☐	☒	A statement on whether measurements were taken from distinct samples or whether the same sample was measured repeatedly
☐	☒	The statistical test(s) used AND whether they are one- or two-sided<br>*Only common tests should be described solely by name; describe more complex techniques in the Methods section.*
☒	☐	A description of all covariates tested
☐	☒	A description of any assumptions or corrections, such as tests of normality and adjustment for multiple comparisons
☐	☒	A full description of the statistical parameters including central tendency (e.g. means) or other basic estimates (e.g. regression coefficient) AND variation (e.g. standard deviation) or associated estimates of uncertainty (e.g. confidence intervals)
☐	☒	For null hypothesis testing, the test statistic (e.g. *F*, *t*, *r*) with confidence intervals, effect sizes, degrees of freedom and *P* value noted<br>*Give P values as exact values whenever suitable.*
☒	☐	For Bayesian analysis, information on the choice of priors and Markov chain Monte Carlo settings
☒	☐	For hierarchical and complex designs, identification of the appropriate level for tests and full reporting of outcomes
☒	☐	Estimates of effect sizes (e.g. Cohen's *d*, Pearson's *r*), indicating how they were calculated

Our web collection on statistics for biologists contains articles on many of the points above.

Software and code

Policy information about availability of computer code

Data collection	Next-generation sequencing was performed by a commercial vendor (Genewiz, Illumina MiSeq).<br>Fluorescence microscope (Olympus, IX51) and Spinning Disk Confocal Microscope (Olympus, DSU-IX81) were used to acquire images.<br>Plate readers: Hybrid Multi-Mode Reader (BioTek, Synergy Neo2) and Microplate reader (BMG Labtech, FLUOstar Omega).<br>Personal assay BLItz System (ForteBio) for BLI assays.
Data analysis	MAGeCK (Li et al., 2014) for analyzing screen data.<br>Excel (Microsoft, 2007) and OriginPro (OriginLab, v8.5) for data and statistical analysis.<br>BLItz pro. software (ForteBio, Version 1.1.0.29) for analyzing BLI data.<br>ImageJ (Version 1.52o) for analyzing images.<br>R (v4.1.1), seqinr (v4.2-8), BALCONY (v0.2.10 packages), and ComplexHeatmap (v2.8.0) for sequence analysis.<br>PyMol (v2.4.1) for structural analysis.

For manuscripts utilizing custom algorithms or software that are central to the research but not yet described in published literature, software must be made available to editors and reviewers. We strongly encourage code deposition in a community repository (e.g. GitHub). See the Nature Portfolio guidelines for submitting code & software for further information.

Data

Policy information about availability of data

All manuscripts must include a data availability statement. This statement should provide the following information, where applicable:

- Accession codes, unique identifiers, or web links for publicly available datasets
- A description of any restrictions on data availability
- For clinical datasets or third party data, please ensure that the statement adheres to our policy

DiffBase database (https://diffbase.uwaterloo.ca/) for downloading TcdB sequences.
HaploColor algorithm (https://github.com/doxeylab/haploColor) for haplotype visualization.

Human research participants

Policy information about studies involving human research participants and Sex and Gender in Research.

Reporting on sex and gender	N/A
Population characteristics	N/A
Recruitment	N/A
Ethics oversight	N/A

Note that full information on the approval of the study protocol must also be provided in the manuscript.

Field-specific reporting

Please select the one below that is the best fit for your research. If you are not sure, read the appropriate sections before making your selection.

[x] Life sciences   [ ] Behavioural & social sciences   [ ] Ecological, evolutionary & environmental sciences

For a reference copy of the document with all sections, see nature.com/documents/nr-reporting-summary-flat.pdf

Life sciences study design

All studies must disclose on these points even when the disclosure is negative.

Sample size	Data were from at least three independent biological replicates. For organoids and in vivo experiments, the sample sizes were chosen based on previous literatures (PMID: 27680706; PMID: 34145250).
Data exclusions	No data are excluded from analysis.
Replication	To ensure robust reproducibility:<br>Cell-rounding assay and competition assays were repeated at least 3 times;<br>Immunoblot analysis was repeated at least twice;<br>Biolayer interferometry (BLI) assay was repeated at least 3 times;<br>Toxin cell surface binding and immunostaining assay was repeated at least twice;<br>Factor Xa activity assay was repeated for 3 times;<br>Cecum-injection assay was repeated for 3 times;<br>Limited proteolysis assay was repeated for twice;<br>In vivo toxicity assays were repeated for 3 times;<br>All replication attempts were successful and observed consistent patterns.
Randomization	For cell-rounding assay, the phase-contrast images were taken and a zone containing 50~200 cells (~300 × 300 μm) was selected randomly. For in vivo experiments, the mice were grouped for different treatments randomly.
Blinding	The cell-rounding assays, organoids and in vivo experiments were done blindly. For other experiments no blinding was performed. This is because blinding could lead to a risk of mislabeling for samples, for example the toxin fragments with point mutations.

Reporting for specific materials, systems and methods

We require information from authors about some types of materials, experimental systems and methods used in many studies. Here, indicate whether each material, system or method listed is relevant to your study. If you are not sure if a list item applies to your research, read the appropriate section before selecting a response.

Materials & experimental systems

n/a	Involved in the study
	☒ Antibodies
	☒ Eukaryotic cell lines
☒	Palaeontology and archaeology
	☒ Animals and other organisms
☒	Clinical data
☒	Dual use research of concern

Methods

n/a	Involved in the study
☒	ChIP-seq
☒	Flow cytometry
☒	MRI-based neuroimaging

Antibodies

Antibodies used

Mouse monoclonal anti-Actin, Aves Labs, ACT-1010 (Antibody Registry ID: AB_2313504)
Mouse monoclonal anti-HA, BioLegend, 901502 (Clone: 16B12)
Chicken polyclonal anti-HA, AVES Labs, ET-HA100
Mouse monoclonal anti-FLAG, Sigma, F3165 (Clone: M2)
Rabbit monoclonal anti-FLAG, Abcam, ab205606 (Clone number: EPR20018-251)
Mouse monoclonal anti-Rhodopsin (1D4), ThermoFisher, MA1-722 (RRID: AB_325050)
Rabbit polyclonal anti-TFPI, Abcam, ab260042
Chicken polyclonal anti-TcdB, List Bio, 754A
Goat anti-Rabbit IgG Secondary Antibody, Alexa Fluor™ 488, ThermoFisher, # A-11008
Goat anti-Mouse IgG Secondary Antibody, Alexa Fluor™ 546, ThermoFisher, # A-11030

Validation

Antibodies have been validated by manufacturers and their data are satisfactory:
Mouse monoclonal anti-Actin: Species Reactivity: Human, Mouse, Rat; Applications: WB
Mouse monoclonal anti-HA, Species Reactivity: HA tag (peptide CYPYDVPDYASL); Applications: WB, FC, ICC, IP, Purification
Chicken polyclonal anti-HA, Species Reactivity: HA tag (peptide CYPYDVPDYASL); Applications: ELISA, ICC, IHC, IP, WB
Mouse monoclonal anti-FLAG, Species Reactivity: FLAG tag (peptide DYKDDDDK); Applications: WB, IP, IHC, ICC, ELISA, EIA, Flow Cyt
Rabbit monoclonal anti-FLAG, Species Reactivity: FLAG tag (peptide DYKDDDDK); Applications: WB, ICC/IF, Flow Cyt, IHC-P, IP
Mouse monoclonal anti-Rhodopsin (1D4), Species Reactivity: Bovine, Human, Mouse, Non-human primate, Rat; Applications: WB, ICC/IF, Flow Cyt, IHC-P, IP
Rabbit polyclonal anti-TFPI, Species Reactivity: Human; Applications: WB, ICC/IF, IP
Chicken polyclonal anti-TcdB, Species Reactivity: unknown; Applications: WB, ICC/IF, IP

Eukaryotic cell lines

Policy information about cell lines and Sex and Gender in Research

Cell line source(s)

HeLa, ATCC, CCL-2
A549, ATCC, CRM-CCL-185
5637, ATCC, HTB-9
HEK293T, ATCC, CRL-3216
Expi293F, Life Technologies, A14527
The Cas9 expressing cells were generated in the lab (references: PMID: 27680706; PMID: 34145250).
The knockout cells and over-expression cells were generated in the lab (sex according to the wild-type cell lines).

Authentication

Cells were not authenticated in our lab.

Mycoplasma contamination

Cell lines have been tested for mycoplasma contamination before. No recent test and no indication of contamination was observed.

Commonly misidentified lines
(See ICLAC register)

No commonly misidentified cell lines were used.

Animals and other research organisms

Policy information about studies involving animals; ARRIVE guidelines recommended for reporting animal research, and Sex and Gender in Research

Laboratory animals

CD1 mice, Charles River, #022 (8-10 weeks of age, 17 – 20 g bodyweight)
housing conditions: dark/light cycle (12 h), ambient temperature (25 degree) and humidity (30%-40%).

Wild animals

No wild animals were used in this study

Reporting on sex	Cecum injection assay was done in female mice;<br>In vivo toxicity assays were done in both male and female mice.
Field-collected samples	No field-collected samples were used in this study.
Ethics oversight	All animal studies, including euthanasia via carbon dioxide asphyxiation, were conducted according to ethical regulations under protocols approved by the Institute Animal Care and Use Committee (IACUC) at Boston Children's Hospital (18-10-3794R).

Note that full information on the approval of the study protocol must also be provided in the manuscript.
